# Temporal landscape of mitochondrial proteostasis governed by the UPR^mt^

**DOI:** 10.1126/sciadv.adh8228

**Published:** 2023-09-22

**Authors:** Louise Uoselis, Runa Lindblom, Wai Kit Lam, Catharina J. Küng, Marvin Skulsuppaisarn, Grace Khuu, Thanh N. Nguyen, Danielle L. Rudler, Aleksandra Filipovska, Ralf B. Schittenhelm, Michael Lazarou

**Affiliations:** ^1^ Walter and Eliza Hall Institute of Medical Research, Parkville, Victoria, Australia.; ^2^ Department of Biochemistry and Molecular Biology, Biomedicine Discovery Institute, Monash University, Melbourne, Australia.; ^3^ Aligning Science Across Parkinson’s Collaborative Research Network, Chevy Chase, MD 20185, USA.; ^4^ Harry Perkins Institute of Medical Research and ARC Centre of Excellence in Synthetic Biology, Nedlands, Western Australia, Australia.; ^5^ Telethon Kids Institute, Northern Entrance, Perth Children’s Hospital, Nedlands, Western Australia, Australia.; ^6^ Monash Proteomics and Metabolomics Facility, Department of Biochemistry and Molecular Biology, Biomedicine Discovery Institute, Monash University, Melbourne, Australia.; ^7^ Department of Medical Biology, University of Melbourne, Melbourne, Victoria, Australia.

## Abstract

Breakdown of mitochondrial proteostasis activates quality control pathways including the mitochondrial unfolded protein response (UPR^mt^) and PINK1/Parkin mitophagy. However, beyond the up-regulation of chaperones and proteases, we have a limited understanding of how the UPR^mt^ remodels and restores damaged mitochondrial proteomes. Here, we have developed a functional proteomics framework, termed MitoPQ (Mitochondrial Proteostasis Quantification), to dissect the UPR^mt^’s role in maintaining proteostasis during stress. We find essential roles for the UPR^mt^ in both protecting and repairing proteostasis, with oxidative phosphorylation metabolism being a central target of the UPR^mt^. Transcriptome analyses together with MitoPQ reveal that UPR^mt^ transcription factors drive independent signaling arms that act in concert to maintain proteostasis. Unidirectional interplay between the UPR^mt^ and PINK1/Parkin mitophagy was found to promote oxidative phosphorylation recovery when the UPR^mt^ failed. Collectively, this study defines the network of proteostasis mediated by the UPR^mt^ and highlights the value of functional proteomics in decoding stressed proteomes.

## Teaser

Functional proteomics reveals how the UPR^mt^ safeguards mitochondrial proteostasis and metabolism during stress.

## INTRODUCTION

Mitochondrial quality control involves repair and removal processes that maintain mitochondrial health in the face of stress. Failure to maintain mitochondrial health has been implicated in the pathology of several human diseases ranging from neurodegenerative diseases, including Parkinson’s disease and Alzheimer’s disease to diabetes and cancer (*1*–*4*). The fundamental biology of mitochondria, including metabolism and energy generation, is dictated by protein machineries whose proteostasis must be maintained to prevent dysfunction. The mitochondrial unfolded protein response (UPR^mt^) and PINK1/Parkin mitophagy are triggered in response to mitochondrial dysfunction but play opposing roles to restore proteostasis. PINK1/Parkin mitophagy drives the degradation of severely damaged mitochondria through the de novo formation of autophagosomes that encapsulate damaged mitochondria before delivering them to lysosomes for degradation (*5*–*8*). In contrast, the UPR^mt^ is a repair-driven process that induces the transcription of nuclear-encoded factors including chaperones and proteases to repair the protein folding environment of mitochondria (*9*). While both the UPR^mt^ and PINK1/Parkin mitophagy are activated in response to protein folding stress within mitochondria (*10*, *11*), the interplay and cross-talk between these pathways is not well understood.

The UPR^mt^ has been best characterized in the model organism *Caenorhabditis elegans*, which has revealed critical insights into the organismal and cellular roles of the UPR^mt^ (*12*, *13*). In *C. elegans*, the transcription factors activating transcription factor associated with stress-1 (ATFS-1) and defective proventriculus in Drosophila homologue-1 (DVE-1) are key signaling factors for the UPR^mt^ that play several roles to restore mitochondrial health including metabolic rewiring and driving the expression of proteostasis-associated genes (*14*–*17*). In human cells, the orthologue of ATFS-1 was identified to be activating transcription factor 5 (ATF5) (*18*). However, beyond ATF5, there has been considerable evolutionary divergence in UPR^mt^ signaling between *C. elegans* and humans including expansion of transcription factors and additional signaling mechanisms (*19*). During the UPR^mt^ in human cells, the mitochondrial protein DAP3-binding cell death enhancer 1 (DELE1) undergoes protein folding stress–induced cleavage that releases it to the cytosol where it signals the activation of the UPR^mt^ (*20*–*22*). An important component of DELE1 signaling is up-regulated expression of key mammalian UPR^mt^-associated transcription factors including C/EBP Homologous Protein (CHOP) and activating transcription factor 4 (ATF4) (*20*–*22*), of which CHOP is completely absent from the *C. elegans* genome (*23*). Collectively, these transcription factors drive a largely undefined UPR^mt^ program that involves proteases and chaperones to repair mitochondrial damage (*9*, *18*, *24*, *25*). The signaling hierarchy between each transcription factor, in addition to the relative importance of CHOP, ATF4, and ATF5, with respect to whether they act alone or in concert in driving the mammalian UPR^mt^ is unknown.

Analyses of proteostasis during the UPR^mt^ so far have relied on quantitative proteomics, and while these approaches can accurately quantify protein levels and provide important insights (*25*, *26*), they do not capture changes to the functional proteome that are independent of protein quantity. Functional changes include posttranslational modifications, protein-protein interactions, or, most importantly with respect to disruptions in proteostasis, protein folding status. Given this, we have a limited understanding of vulnerabilities within the mitochondrial proteome (mito-proteome) during protein folding stress and the role played by the UPR^mt^ in maintaining proteostasis.

Here, we have developed a functional proteomics framework that enables quantitative analysis of mitochondrial proteostasis. Termed Mitochondrial Proteostasis Quantification (MitoPQ), the experimental pipeline was used to generate a temporal profile of mitochondrial proteostasis before, during, and after recovery from proteostasis stress. Combining MitoPQ with knockout (KO) lines of CHOP, ATF4, and ATF5, or all three, revealed that the UPR^mt^ functions during two distinct phases of proteostasis stress: a protection phase that limits damage, followed by a repair phase that restores proteostasis. Mitochondrial transcription and translation and oxidative phosphorylation (OXPHOS) metabolism were identified as being highly vulnerable to protein folding stress, with complex I having a high reliance on the UPR^mt^ for protection and repair. Through transcriptomics and MitoPQ, we find that CHOP, ATF4, and ATF5 function in concert by driving independent arms of the UPR^mt^ program to nonredundantly protect and repair mitochondrial proteostasis. Assessment of the interplay between PINK1/Parkin mitophagy and the UPR^mt^ identified a unidirectional signaling relationship between the pathways and a role for the UPR^mt^ in sustaining PINK1/Parkin mitophagy activity during the repair phase. We also identify PINK1/Parkin mitophagy as a secondary response to proteostasis stress that serves to address mitochondrial dysfunction when the UPR^mt^ fails or is overwhelmed. Overall, our work defines the role of the UPR^mt^ in protecting and repairing mitochondrial metabolism while providing a functional proteomics framework for the analysis of stressed proteomes.

## RESULTS

### MitoPQ is a quantitative proteomics framework for the analysis of mitochondrial proteostasis

**Fig. 1.**
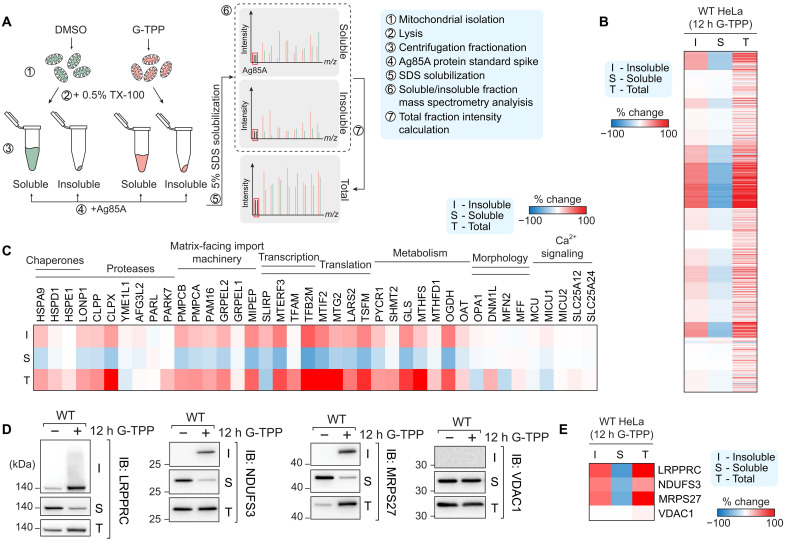
Developing an experimental pipeline to analyze endogenous mitochondrial proteostasis. (**A**) Workflow for proteostasis quantification of mitochondria. TX-100, Triton X-100. (**B**) Heatmap analysis of mitochondrial protein solubility changes following 12 hours of G-TPP treatment. (**C**) Heatmap of protein solubility changes grouped by mitochondrial process. (**D**) Immunoblot (IB) validation of solubility trends identified using MitoPQ analysis. (**E**) Heatmap of solubility changes of proteins validated by immunoblot. Data in (B), (C), and (E) represent mean data calculated from three independent experiments.

The UPR^mt^ is thought to play a fundamental role in mitochondrial proteostasis (*27*); yet, despite this, it remains unknown to what extent the UPR^mt^ program protects or repairs proteostasis during stress, while the individual roles of the key UPR^mt^ transcription factors CHOP, ATF4, and ATF5 have not been explored. To address these gaps, we established MitoPQ, a quantitative proteomics framework designed to measure mitochondrial protein solubility. The workflow, summarized in Fig. 1A, uses isolated mitochondria and involves extracting soluble and insoluble proteins based on their separation with 0.5% Triton X-100. Detergent soluble and insoluble protein fractions are then extracted using 5% SDS, sonication, and low pH buffers (<pH 3). To each fraction, we add a standardized amount of the bacterial protein Ag85A from *Mycobacterium tuberculosis* that enables a relative comparison of peptide intensities in soluble and insoluble mitochondrial fractions, and the calculation of a relative total amount of protein that is used to measure shifts in protein solubility as a percentage of the total fraction.

To validate the utility of MitoPQ in measuring mitochondrial proteostasis, we performed MitoPQ analysis on HeLa cells exposed to a mitochondrial protein folding stress. HeLa cells were treated for 12 hours with Gamitrinib-triphenylphosphonium (G-TPP), a mitochondrial specific heat shock protein 90 (HSP90) inhibitor that induces mitochondrial protein misfolding (*11*, *26*). A total of 995 mitochondrial proteins were identified across the soluble and insoluble fractions (Fig. 1B). A global clustered heatmap analysis identified protein groups that displayed varied trends in changes to total protein levels and solubility following protein folding stress (Fig. 1B). This included 221 mitochondrial proteins that became more insoluble following G-TPP treatment (>25% shift to the insoluble fraction) and 109 mitochondrial proteins whose solubility did not change but total protein levels did change, demonstrating varied changes to proteostasis across the mito-proteome (Fig. 1B). Analysis of select mitochondrial machineries revealed that mitochondrial transcription and translation machineries, along with key metabolism-associated factors including glutaminase (GLS), methenyl-THF synthetase (MTHFS), and 2-oxoglutarate dehydrogenase complex component E1 (OGDH), were acutely sensitive to solubility collapse following proteostasis stress (Fig. 1C). In contrast, mitochondrial morphology and Ca^2+^ signaling proteins were largely unaffected, indicating that sensitivity to proteostatic stress differs across the functional components of the mito-proteome (Fig. 1C). To confirm the accuracy of MitoPQ, we analyzed the solubility and total protein trends in cells after 12 hours of dimethyl sulfoxide (DMSO) or G-TPP treatment by Western blotting (Fig. 1D). Protein level trends in total, soluble, and insoluble fractions of select aggregating proteins [leucine-rich PPR motif-containing protein (LRPPRC), NADH dehydrogenase [ubiquinone] iron-sulfur protein 3 (NDUFS3), and mitochondrial ribosomal protein S27 (MRPS27)] and a soluble control [voltage-dependent anion-selective channel protein 1 (VDAC1)] assessed by immunoblotting (Fig. 1D) mirrored those produced by MitoPQ analysis (Fig. 1E), confirming the accuracy of the MitoPQ framework in quantifying changes to mitochondrial protein solubility.

### CHOP, ATF4, and ATF5 play essential roles to protect and repair mitochondrial proteostasis

**Fig. 2.**
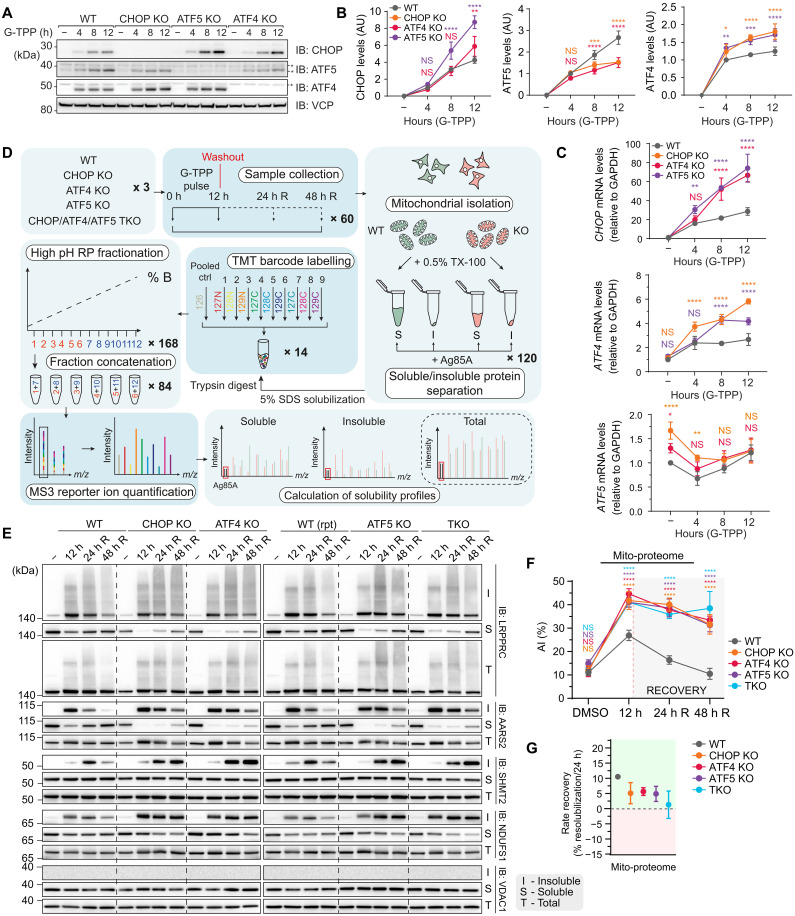
Independent signaling pathways driven by CHOP, ATF4, and ATF5 are required for UPR^mt^-driven proteostasis protection and repair. (**A** to **C**) WT, CHOP KO, ATF4 KO, and ATF5 KO cells were treated with G-TPP, and transcription factor expression was analyzed by immunoblot (A) and quantified (B). AU, arbitrary units. mRNA expression of each transcription factor was analyzed by qRT-PCR and quantified (C). (**D**) Experimental design. RP, reversed phase. (**E**) Immunoblot validation of solubility trends identified in the experiment outlined in (D) using MitoPQ analysis. rpt, repeat. (**F**) Plot of mito-proteome aggregation indices (AIs) in WT, CHOP KO, ATF4 KO, ATF5 KO, and TKO samples. (**G**) Plot of resolubilization rates for WT, CHOP KO, ATF4 KO, ATF5 KO, and TKO cells across the mito-proteome (linked to data in table S1). Data in (B), (C), (F), and (G) are mean ± SD from three independent experiments. *P < 0.05, **P < 0.005, ***P < 0.001, and ****P < 0.0001 [two-way analysis of variance (ANOVA)]. NS, not significant. R, recovery.

Having established and validated MitoPQ, we next applied it to the fundamental question of how the UPR^mt^ maintains mitochondrial proteostasis through addressing the role played by UPR^mt^ transcription factors. HeLa cell KO lines of key UPR^mt^ transcription factors CHOP, ATF4, and ATF5, along with a triple KO (TKO) line lacking all three transcription factors, were generated using CRISPR-Cas9 (ATF4 and ATF5), or TALEN (CHOP) mediated gene editing (fig. S1A and table S3). First, we explored transcription factor induction in each KO line since previous reports have indicated that ATF4 is an upstream regulator of CHOP and ATF5 during the integrated stress response (*28*, *29*). Immunoblot and mRNA analysis of transcription factor induction following G-TPP treatment of KO lines showed that, in contrast to the canonical integrated stress response in which ATF4 is the master regulator (*28*, *29*), each of CHOP, ATF4, and ATF5 was activated independently of each other during the UPR^mt^ (Fig. 2, A to C). ATF5 induction occurred post-translationally (Fig. 2, A to C), consistent with previous observations (*18*). The loss of CHOP and ATF4 affected ATF5 mRNA levels basally but not during G-TPP stress (Fig. 2C), while ATF5 protein levels were partially lower in the absence of CHOP or ATF4 (Fig. 2, A and B). Overall, these results demonstrate that CHOP, ATF4, and ATF5 can be independently expressed during the UPR^mt^ and may therefore represent independent signaling arms of the UPR^mt^ program.

We next addressed the contribution of each transcription factor to the protection and repair of mitochondrial proteostasis during stress. The MitoPQ framework was coupled with tandem mass tags (TMT) multiplex barcode labeling and applied to transcription factor KO lines treated with G-TPP. Cells were analyzed at four different time points, including a 12-hour DMSO treatment control, a 12-hour G-TPP treatment to assess UPR^mt^-mediated protection against proteostasis damage, and 24- and 48-hour G-TPP washout time points, termed recovery, to assess UPR^mt^-mediated proteostasis repair. A schematic summary of the experimental workflow is shown in Fig. 2D. Temporal solubility profiles were calculated for 884 mitochondrial proteins across the entire dataset. Principal component analyses showed highly reproducible, time-dependent proteostasis patterns across each cell line, with baseline DMSO samples clustering with recovery samples in wild-type (WT) cells, but not in KO lines (see “DMSO” and “48 h R” in fig. S1, B and C). Immunoblot analysis of select proteins identified by MitoPQ as highly aggregating (LRPPRC and AARS2), mildly aggregating (NDUFS1 and SHMT2), or completely soluble (VDAC1), validated the temporal proteostasis profiles generated by MitoPQ analysis (Fig. 2E), across multiple KO clones (fig. S2).

Next, we compared the global proteostasis profiles across all KO cell lines and the WT control by calculating an aggregation index that represents the global average of protein aggregation in each cell line at each time point (Fig. 2F). The aggregation index was highest in each cell line at the 12-hour G-TPP treatment time point (Fig. 2F), with CHOP, ATF4, and ATF5 KOs displaying significantly higher levels of aggregation than the WT control (Fig. 2F). Analysis of the recovery time points (24-hour R and 48-hour R) in WT cells showed that the aggregation index decreased at 24 hours, and returned to the DMSO baseline by 48 hours (Fig. 2F), demonstrating an almost complete recovery from the protein folding stress. In contrast, each transcription factor KO cell line showed little reduction in the aggregation index at 24-hour recovery, and minimal reduction by 48 hours (Fig. 2F). The apparent lack of recovery in KO lines may be influenced by their high aggregation load at the 12-hour treatment time point. To overcome this, we assessed global recovery rates as a percentage of aggregation reduction over a 24-hour period (Fig. 2G and table S1) and found that all KO lines had a ~50% or greater reduction in their rate of recovery relative to the WT control. These results demonstrate that the UPR^mt^ functions during two distinct phases of mitochondrial protein folding stress: (i) A protection phase that occurs during the insult to limit proteostasis damage and (ii) a repair phase that occurs following the removal of the insult to restore proteostasis.

We noted that the TKO line did not show an additive proteostasis defect, either during the protection or repair phases of the UPR^mt^ (Fig. 2, F and G). However, given that the aggregation index is a measure of average protein aggregation, we assessed whether TKO cells had a higher proportion of very highly aggregating proteins. TKO cells did not display an increased level of highly aggregating proteins that we classified as having >50% solubility shift (fig. S3). These results demonstrate that each of the CHOP, ATF4, and ATF5 signaling arms of the UPR^mt^ play equally important roles in protecting and repairing mitochondrial proteostasis.

### Mitochondrial processes are differentially affected by the loss of UPR^mt^ function

**Fig. 3.**
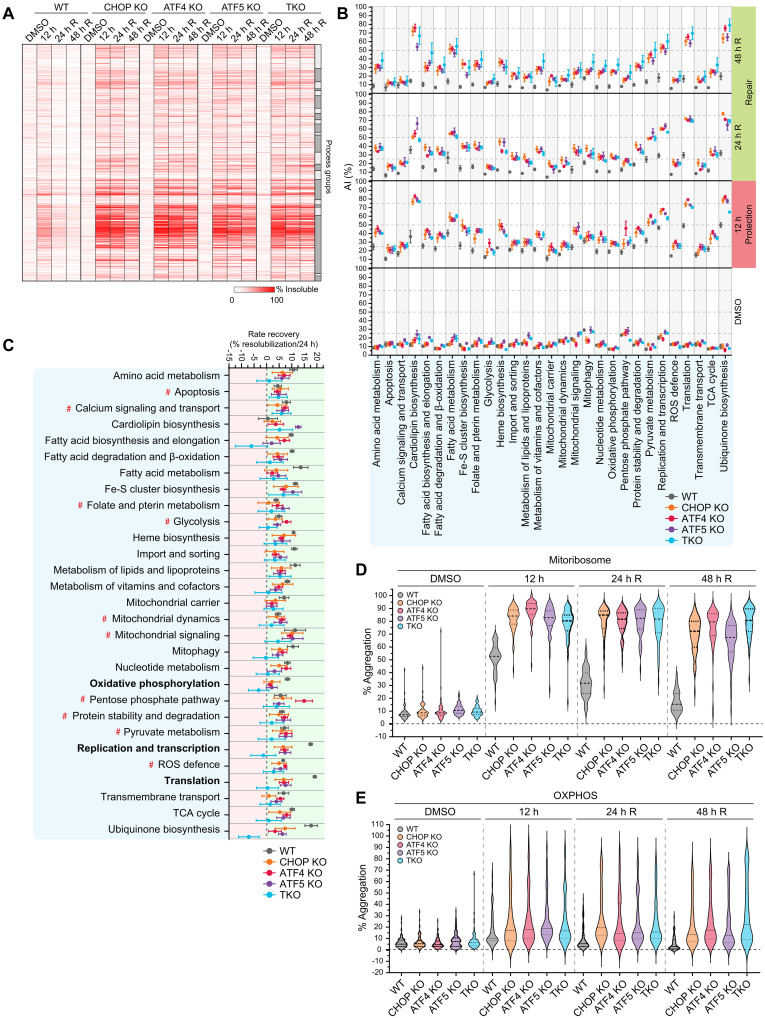
Each CHOP, ATF4, and ATF5 signaling pathway functions nonredundantly in driving UPR^mt^-mediated proteostasis protection and repair. (**A**) Heatmap analysis clustered by mitochondrial process of solubility trends in WT, CHOP KO, ATF4 KO, ATF5 KO, and TKO cells across the indicated time points. Alternating light and dark gray boxes correspond to individual process groups. (**B**) Aggregation indices (AI) of WT, CHOP KO, ATF4 KO, ATF5 KO, and TKO cells were calculated and graphed for each mitochondrial process group (linked to data in table S2). (**C**) Resolubilization rates for WT, CHOP KO, ATF4 KO, ATF5 KO, and TKO cells were calculated and graphed for each mitochondrial process group. (**D** and **E**) Mean % aggregation of proteins belonging to the mitochondrial ribosome (D) or the OXPHOS machinery (E) was graphed by violin plot. Solid lines, median; dotted lines, quartiles. Data in (A), (D), and (E) represent mean data calculated from three independent experiments. Data in (B) and (C) are mean ± SD from three independent experiments. R, recovery.

The global analysis in Fig. 2F provided a broad overview of mitochondrial protein aggregation. However, we also wanted to gain information about specific mitochondrial processes to assess their sensitivity to aggregation in the presence/absence of a functional UPR^mt^ program. In addition, we wanted to address whether CHOP, ATF4, or ATF5 have any selectivity toward the protection or repair of proteins associated with specific mitochondrial processes. A broad heatmap analysis was conducted on mitochondrial proteins grouped according to their functional processes using a list curated by Kuznetsova *et al.* (*30*) (Fig. 3A). Following 12 hours of G-TPP treatment, the heatmap revealed clusters of proteins with elevated aggregation in WT cells (Fig. 3A) that were further aggregated in UPR^mt^-deficient transcription factor KO lines. The highly aggregating clusters displayed a resistance to repair in the absence of the UPR^mt^ (24- and 48-hour recovery; Fig. 3A). The heatmap analysis indicates that proteins from certain mitochondrial processes are sensitive to protein folding stress and have a reliance on the UPR^mt^ for their proteostasis.

To clearly identify and characterize the mitochondrial processes that belonged to the aggregation sensitive protein clusters in Fig. 3A, an aggregation index was calculated for each functional process group and plotted across the protection and recovery phases (Fig. 3B and table S2). In WT cells, all mitochondrial process groups displayed an elevated aggregation index following 12 hours of G-TPP treatment except for apoptosis, glycolysis, mitochondrial carrier, and transmembrane transport (Fig. 3B). High aggregation index values (>40%) at 12 hours of G-TPP treatment were observed for metabolism related processes including fatty acid metabolism and ubiquinone biosynthesis, in addition to replication and transcription, and translation (Fig. 3B), indicating high sensitivity of these mitochondrial processes to proteostasis stress. In transcription factor KO lines, all mitochondrial process groups had aggregation indexes that were above the WT control to varying degrees, with metabolism related processes such as Fe-S biosynthesis, cardiolipin biosynthesis, ubiquinone biosynthesis, and translation showing a very high reliance on the UPR^mt^ during the protection phase (Fig. 3B).

Analysis of the 24- and 48-hour recovery time points, representing the proteostasis repair phase of the UPR^mt^, showed that WT cells returned to baseline aggregation indexes by 48-hour recovery across all process groups (Fig. 3B). In contrast, with the exception of apoptosis and calcium signaling and transport, aggregation indexes remained high in all transcription factor KO lines demonstrating widespread defects in proteostasis repair. Recovery rates were also calculated for each mitochondrial process group to further assess UPR^mt^-mediated proteostasis repair (Fig. 3C and table S1). Unexpectedly, this analysis revealed that multiple process groups in transcription factor KO cells had similar recovery rates to WT, including folate and pterin metabolism and glycolysis (see process groups marked by # in Fig. 3C), demonstrating a reliance on the UPR^mt^ for protection (Fig. 3B) but not repair. In contrast, several mitochondrial processes required the UPR^mt^ for both protection and repair, and these included oxidative phosphorylation, replication and transcription, and translation process groupings (Fig. 3C, highlighted in bold text). The TKO line had broadly similar protection and repair defects to the single KO lines (Fig. 3, A to C, and tables S1 and S2), although decreased recovery rates were observed for fatty acid metabolism, ubiquinone biosynthesis, replication and transcription, and translation (Fig. 3C), indicating partial redundancy of the transcription factors for these mitochondrial processes. Overall, CHOP, ATF4, or ATF5 did not show a preference in protecting or repairing specific mitochondrial processes, with all three transcription factors playing equally important roles in maintaining proteostasis. In addition, we find a divergence in mitochondrial processes in terms of their requirement for the CHOP-, ATF4-, and ATF5-driven UPR^mt^ program, with some requiring it solely for protection from stress (e.g., glycolysis), while the majority was reliant on the UPR^mt^ for both protection and repair.

### The UPR^mt^ protects and repairs complex I to maintain OXPHOS activity

**Fig. 4.**
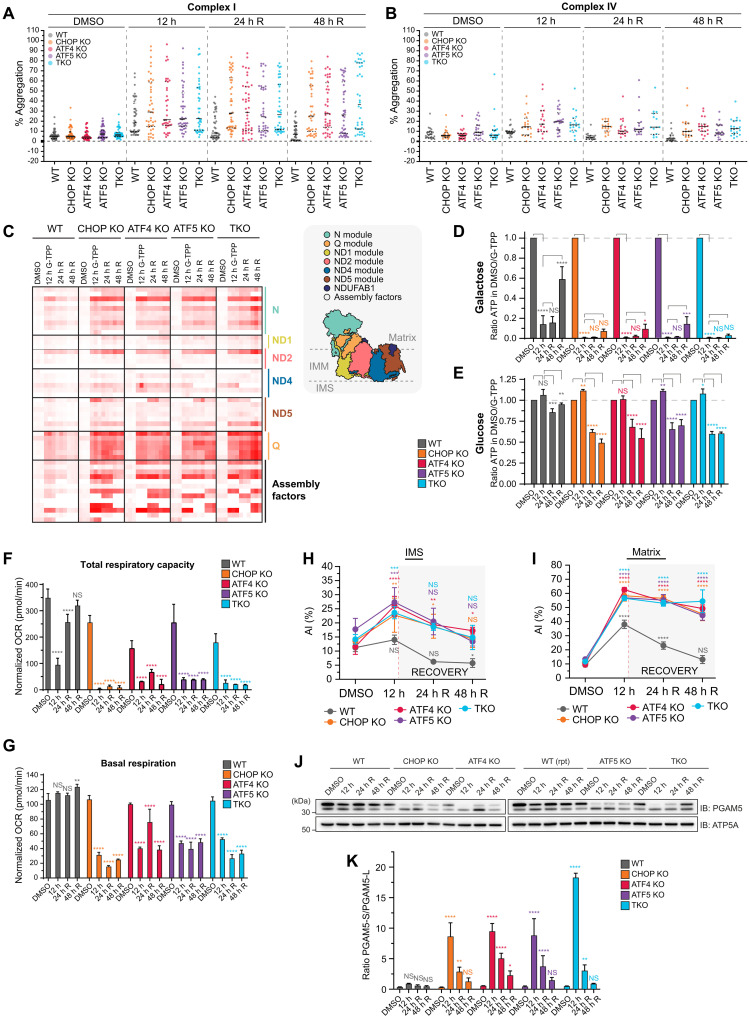
The UPR^mt^ protects and repairs OXPHOS solubility and activity during proteostasis stress. (**A** and **B**) Violin plots of the mean % aggregation of proteins comprising complex I (A) and complex IV (B) in WT, CHOP KO, ATF4 KO, ATF5 KO, and TKO cells at the indicated time points. (**C**) Heatmap of the mean % aggregation of proteins comprising complex I, labeled according to complex I sub-module localization in each cell line at the indicated time points. (**D** and **E**) Ratios of cellular ATP levels in each cell line treated in either galactose-based (D) or glucose-based (E) medium were calculated relative to DMSO levels at the indicated time points and graphed. (**F** and **G**) Oxygen consumption rates (OCRs; in picomol per minute) of mitochondria isolated from WT, CHOP KO, ATF5 KO, ATF4 KO, and TKO samples collected at the indicated time points were calculated using a Seahorse (Agilent) analyzer. OCR values of untreated cells were analyzed at each time point and used to normalize OCR values collected at each day of the experimental time course. Normalized OCR values were graphed, and the total respiratory capacity (F) and basal respiration (G) were calculated and graphed for each sample. (**H** and **I**) Aggregation indices (AI) of intermembrane space (IMS)–localized (H) or matrix-localized (I) proteins in each cell line were calculated and graphed. (**J** and **K**) Uncleaved and cleaved PGAM5 levels were analyzed by immunoblot of mitochondria (J) isolated from WT, CHOP KO, ATF4 KO, ATF5 KO, and TKO cells at the indicated time points. Ratios of cleaved/uncleaved PGAM5 were calculated within each sample and graphed (K). Data in (A) to (C) represent mean data calculated from three independent experiments. Data in (D) to (I) and (K) represent mean ± SD from three independent experiments. *P < 0.05, **P < 0.005, ***P < 0.001, and ****P < 0.0001 {two-way ANOVA, relative to DMSO control [(F) to (I) and (K)]}. R, recovery.

OXPHOS and translation were major mitochondrial process groups that were found to rely on the UPR^mt^ for their protection and repair (Fig. 3). These fundamental mitochondrial processes consist of protein complexes that drive their activity. For example, the oxidative phosphorylation process group consists of multiple machineries of the electron transport chain (complexes I to IV) in addition to the adenosine 5′-triphosphate (ATP) synthase complex (complex V). We asked whether all components of the translation machinery and all complexes of OXPHOS machineries were equally disrupted by protein folding stress and to what degree the UPR^mt^ provides protection and repair. The mitoribosome (94% of subunits identified by MitoPQ) displayed broad aggregation across all protein components belonging to both the 39*S* large subunit and the 28*S* small subunit (Fig. 3D). All proteins belonging to both the 39*S* and 28*S* subunits of the mitoribosome underwent proteostasis repair in WT cells but not in UPR^mt^-defective KO lines (Fig. 3D), which was consistent with their impaired recovery rates (Fig. 3C). In contrast to the mitoribosome (Fig. 3D), aggregation analysis of the entire OXPHOS machinery (74% of subunits identified by MitoPQ) showed only a moderate median level of aggregation, but a large upper tail of highly aggregating proteins was observed (Fig. 3E), revealing that a specific subset of OXPHOS proteins are highly sensitive to aggregation. To identify the highly aggregating OXPHOS proteins, violin plots were separated out by individual OXPHOS complexes (complexes I to V) (Fig. 4, A and B, and fig. S4, A to C). This analysis revealed that in both WT and transcription factor KO cells, the highly aggregating OXPHOS proteins were primarily complex I subunits (compare Fig. 4, A with B, and fig. S4, A to C). In UPR^mt^-defective cells, further aggregation of complex I subunits was observed relative to WT controls (Fig. 4A), along with additional aggregation of complexes II to V subunits that were otherwise not strongly aggregated in WT cells (Fig. 4B and fig. S4, A to C). Collectively, the KO lines displayed a failure to both protect and repair complex I proteostasis (Fig. 4A), with the strongest defect observed during the repair phase of the UPR^mt^. Overall, this analysis identifies complex I as a vulnerable component of the OXPHOS machinery that is highly reliant on the UPR^mt^ for its protection and repair during stress. Combined with the observation that the UPR^mt^ also strongly protects and repairs processes that support OXPHOS (cardiolipin biosynthesis, fatty acid metabolism, ubiquinone biosynthesis, and transcription/translation; Fig. 3), we conclude that the UPR^mt^ plays a fundamental role in maintaining OXPHOS metabolism during proteostasis stress.

We noted that complex I subunit aggregation did not display the same whole-complex aggregation trend observed with the mitoribosome (compare Fig. 3E with Fig. 3D). Given that complex I consists of distinct assembly modules (*31*), the module locations of aggregating subunits were mapped onto a heatmap to provide a spatial understanding of the aggregation-sensitive areas of complex I (Fig. 4C). In WT cells, subunits with elevated aggregation were localized primarily in the matrix-facing N- and Q-modules involved in electron transfer (Fig. 4C). Interestingly, transcription factor KO lines showed elevated aggregation of additional membrane-bound complex I subunits belonging to the NADH-ubiquinone oxidoreductase chain 1 (ND1) and ND5 modules involved in proton pumping (*32*), demonstrating a more widespread collapse of complex I proteostasis in the absence of each signaling arm of the UPR^mt^ (Fig. 4C).

To investigate the functional importance of UPR^mt^-mediated protection and repair of OXPHOS proteostasis, and simultaneously assess the accuracy of MitoPQ in determining the functional mito-proteome, we analyzed OXPHOS metabolic activity. First, ATP levels were measured in cells cultured in galactose medium for a period of time to drive OXPHOS-dependent ATP production (Fig. 4D and fig. S4E). Relative to the WT control, all UPR^mt^-defective KO lines had almost undetectable levels of ATP during protein folding stress (Fig. 4D), and they also failed to restore ATP levels following 24- and 48-hour recovery. Indeed, such was the severity of mitochondrial dysfunction that KO lines cultured in glucose also displayed significantly reduced levels of ATP during the recovery phase of G-TPP treatment (Fig. 4E). To directly measure OXPHOS activity, complex I stimulated oxygen consumption rates were analyzed using isolated mitochondria (Fig. 4, F and G, and fig. S4D). In WT cells, there was a robust recovery of total respiratory capacity within 24 hours of stress removal (Fig. 4F), while basal respiration remained undisrupted across all time points (Fig. 4G). In contrast, all UPR^mt^-defective KO lines had severely defective respiratory capacity that was unrecoverable (Fig. 4F), highlighting the importance of each of CHOP, ATF4, and ATF5 UPR^mt^ arms. Overall, these results reveal the fundamentally important role that the UPR^mt^ plays in maintaining OXPHOS metabolic activity during protein folding stress by protecting and repairing its proteostasis. The results also highlight the utility of the MitoPQ framework in measuring the functional mito-proteome.

### The UPR^mt^ is dispensable for intermembrane space proteostasis repair

Inhibition of mitochondrial HSP90 using G-TPP provides a protein folding stress that originates in the mitochondrial matrix. We hypothesized that failure to contain a protein folding stress originating from the matrix, such as when the UPR^mt^ is defective or overwhelmed, can result in the stress extending to the intermembrane space (IMS) compartment of mitochondria. To test this hypothesis, we compared the levels of protein aggregation in the matrix and IMS compartments of mitochondria between WT controls and UPR^mt^-defective KOs (Fig. 4, H and I). This analysis showed that indeed, when the UPR^mt^ is defective, a protein folding stress originating in the matrix can affect proteostasis in the IMS compartment (Fig. 4H). However, in contrast to the matrix compartment in which UPR^mt^-deficient cells showed little to no evidence of repair (Fig. 4I), the IMS compartment showed recovery that reached close to the DMSO-treated control in UPR^mt^-deficient cells (Fig. 4H).

The cleavage and release of DELE1 from the IMS by the stress-activated protease OMA1 was identified as a key step of UPR^mt^ signaling (*20*, *21*). OMA1 also cleaves the serine/threonine phosphatase phosphoglycerate mutase 5 (PGAM5) (*33*, *34*). Therefore, as an alternative readout of stress within the IMS, we assessed the ratio of inner membrane–localized PGAM5-long (PGAM5-L) and cleaved PGAM5-short (PGAM5-S). Minimal IMS-based stress was observed in WT cells based on PGAM5 cleavage analysis (Fig. 4, J and K). In contrast, all KO lines displayed significant levels of stress (Fig. 4, J and K). A further significant increase in IMS stress was observed in TKO cells relative to single transcription factor KOs (Fig. 4, J and K), indicating some redundancy in the roles of each CHOP, ATF4, and ATF5 in IMS protection. Interestingly, there was a robust recovery of the PGAM5-L/PGAM5-S ratio in all KO lines (Fig. 4, J and K). The UPR^mt^ therefore plays an important role to protect the IMS from proteostasis stress originating in the matrix but is dispensable for recovery from this stress.

### The UPR^mt^ is required for restoration of mitochondrial networks

**Fig. 5.**
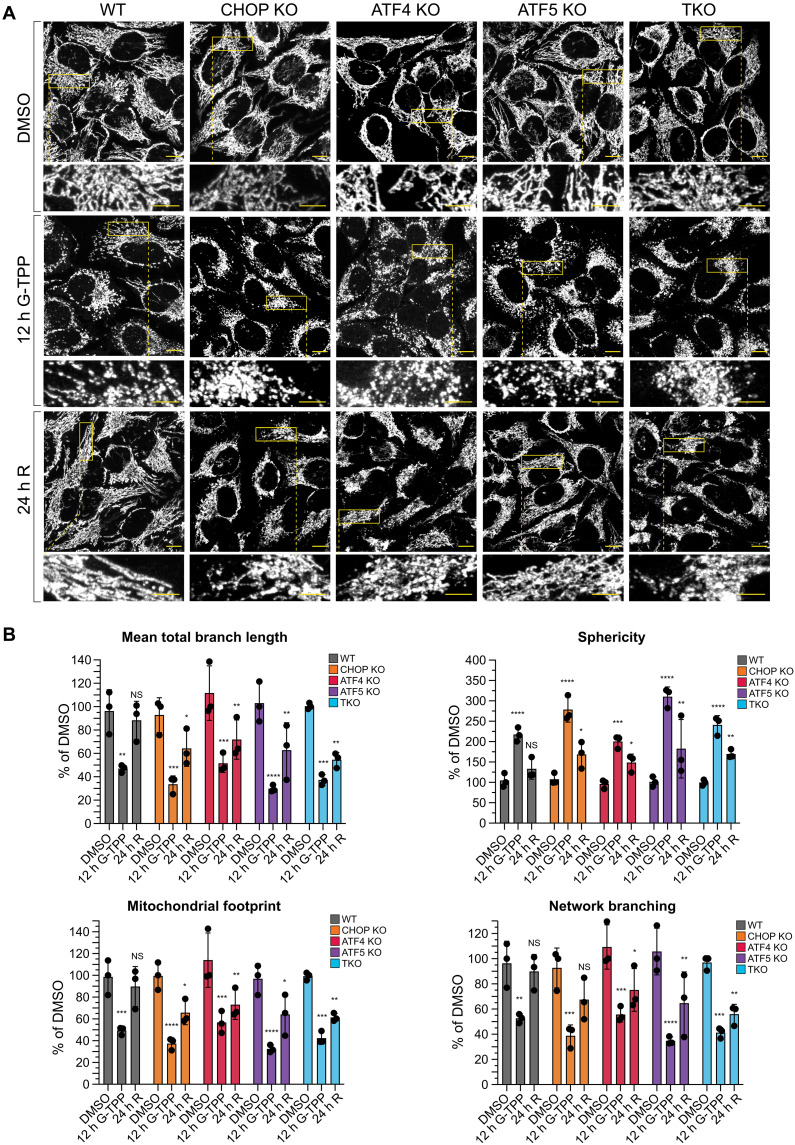
CHOP, ATF4, and ATF5 are required for the restoration of mitochondrial morphology following proteostasis stress. (**A** and **B**) WT, CHOP KO, ATF4 KO, ATF5 KO, and TKO cells analyzed at the indicated time points were immunostained for TOM20 and SDHA (A). TOM20 and SDHA channel signals were combined, and mitochondrial morphology was analyzed using the Mitochondrial Analyzer plugin in Fiji (v2.9.0) (B). Data in (A) are representative images from one experiment, and data in (B) represent mean ± SD from three independent experiments. *P < 0.05, **P < 0.005, ***P < 0.001, and ****P < 0.0001 (two-way ANOVA, relative to DMSO control). Representative images from individual TOM20 and SDHA channels are located in fig. S5. R, recovery. Scale bars, 10 μm overview, 5 μm zoom.

Mitochondrial morphology is linked to mitochondrial stress. For example, mitochondrial network adaptations to stress include hyperfusion or fragmentation (*35*, *36*). We therefore asked how mitochondrial networks adapt to proteostasis stress in the presence or absence of a functional UPR^mt^. WT and transcription factor KO lines were either untreated, treated with G-TPP for 12 hours, or allowed to recover for 24 hours following G-TPP treatment (Fig. 5). Immunofluorescence signals from the outer membrane protein translocase of the outer membrane 20 (TOM20) and the matrix protein succinate dehydrogenase complex flavoprotein subunit A (SDHA) (fig. S5) were combined and used to assess overall mitochondrial morphology and networking using Mitochondria Analyzer (Fig. 5, A and B) (*37*). Upon G-TPP treatment, mitochondria became highly fragmented in all cell lines as evidenced by decreased branch length, decreased network branching, a smaller mitochondrial footprint, and increased sphericity (Fig. 5, A and B). However, while the transcription factor KOs have higher levels of mitochondrial protein aggregation at 12 hours of G-TPP treatment (Fig. 5, A and B), their network fragmentation was not too dissimilar from the WT control (Fig. 5, A and B). This indicates that the proteostasis protection phase of the UPR^mt^ does not strongly influence mitochondrial networking during proteostasis stress. Following 24-hour recovery, WT cells restored their mitochondrial networks back to the levels observed in the DMSO control, whereas in contrast, all transcription factor KOs failed to restore mitochondrial networking to baseline levels, although some degree of network recovery was observed (Fig. 5, A and B). Taken together, these results indicate that mitochondrial proteostasis and mitochondrial networking are associated, and they highlight the importance of a functional UPR^mt^ program for restoration of mitochondrial networks following proteostasis stress.

### CHOP, ATF4, and ATF5 govern common processes via both unique and overlapping genetic programs

**Fig. 6.**
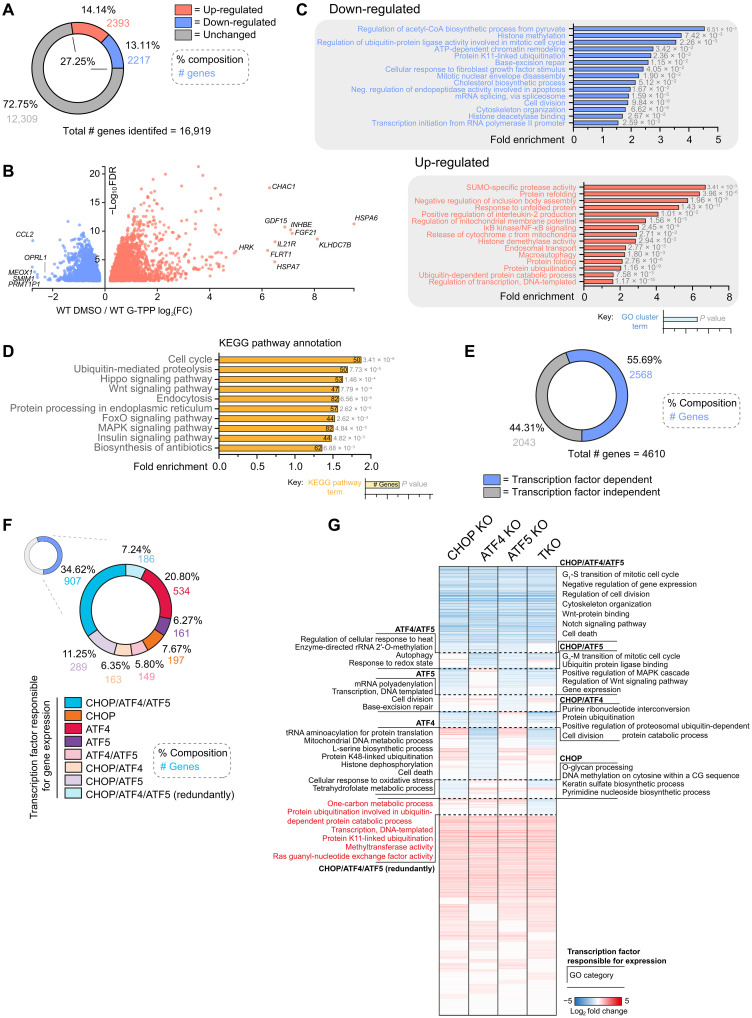
CHOP, ATF4, and ATF5 exert a mosaic pattern of regulatory genetic control to drive the UPR^mt^. (**A**) Pie chart of the percentage and number of genes in the transcriptome significantly altered or unchanged with UPR^mt^ induction. (**B**) Genes significantly up-regulated or down-regulated across the transcriptome in WT cells treated with G-TPP for 12 hours relative to WT cells treated with DMSO for 12 hours were defined as the WT UPR^mt^ transcriptome and graphed. The top 10 up-regulated and top 5 down-regulated genes in the WT UPR^mt^ transcriptome are labeled in (B). FC, fold change. (**C**) Clustered gene ontology (GO) analysis of gene groups up-regulated or down-regulated in expression in the UPR^mt^ transcriptome. Gene clusters have been labeled using the most significantly enriched individual GO category in each cluster group. CoA, coenzyme A; IκB, inhibitor of nuclear factor κB; NF-κB, nuclear factor κB; SUMO, small ubiquitin-like modifier. (**D**) KEGG pathway analysis of WT UPR^mt^ transcriptome trends. MAPK, mitogen-activated protein kinase. (**E**) Pie chart of UPR^mt^ transcriptome genes that were unchanged (“transcription factor independent”) or significantly decreased in expression in either CHOP KO, ATF4 KO, ATF5 KO, or TKO cells treated with G-TPP for 12 hours (“transcription factor dependent”). (**F**) Compositional breakdown displayed by pie chart of the overlapping regulatory patterns of CHOP, ATF4, and ATF5 in the transcription factor–dependent portion of the UPR^mt^ transcriptome. (**G**) Heatmap analysis of gene expression trends across the UPR^mt^ transcriptome in each labeled cell line treated with G-TPP for 12 hours, calculated relative to gene expression in WT cells treated with G-TPP for 12 hours. Significantly enriched GO categories for each regulatory subgroup are labeled along the corresponding section of the heatmap. Data in (A) to (G) represent mean data calculated from three independent experiments. GO analysis in (C) and (G) was performed using the Biological Process and Molecular Function subcategories in Database for Annotation, Visualization, and Integrated Discovery (DAVID) v6.8 (*85*). KEGG pathway analysis in (D) was performed using ShinyGO V0.75 (*86*).

Given that the loss of either CHOP, ATF4, or ATF5 resulted in near identical proteostasis and mitochondrial network defects (Figs. 2 to 5), we asked whether each transcription factor functioned via identical or independent transcriptional programs. To address this, we conducted transcriptome analyses of cells treated with G-TPP for 12 hours. In WT cells, the overall UPR^mt^ program significantly altered the expression 4610 genes (~27% of the detected transcriptome), with similar numbers of up-regulated and down-regulated genes (Fig. 6A), but the highest magnitude of change was observed for up-regulated genes (Fig. 6B). Up-regulated genes were associated with gene ontology (GO) processes linked to protein refolding, response to unfolded protein, and histone demethylase activity (Fig. 6C, red), while down-regulated genes were linked to histone methylation, transcription, and cell cycle (Fig. 6C, blue). Pathway analysis of the transcriptome using KEGG identified cell cycle, ubiquitin-mediated proteolysis, and Wnt signaling, among others, as UPR^mt^-regulated pathways (Fig. 6D). Wnt signaling has been linked to cell-to-cell communication of the UPR^mt^ in *C. elegans* (*38*), and may represent an evolutionary conserved node of the UPR^mt^ program. A more detailed analysis of Wnt signaling pathways revealed diverse gene expression patterns of up-regulated and down-regulated factors (fig. S6A), with the Wnt/Ca^2+^ signaling pathway showing an overall up-regulation. Given the precedence of Wnt signaling in *C. elegans*, the link between the mammalian UPR^mt^ and Wnt is an interesting area for future exploration. To address whether the transcriptional changes resulted directly from UPR^mt^ signaling, or from pleiotropic signals arising from mitochondrial dysfunction, we analyzed the transcriptome of UPR^mt^ signaling deficient DELE1 KO cells (*20*, *21*). An almost complete ablation of the UPR^mt^ transcriptional program was observed in DELE1 KOs (fig. S6, B and C). In addition, analysis of WT cells showed that the canonical UPR^mt^ factors HSPD1, HSPE1, HSP60, and LONP1 were up-regulated at both the mRNA and protein level, and up-regulation of CLPP and HSPA9 were observed at the protein level (Fig. 1C and fig. S6, D and E). Together, these results confirm that the cellular pathways and processes identified above were a direct consequence of UPR^mt^ signaling originating from mitochondria.

We next analyzed the transcriptome of transcription factor KO lines to understand their contribution to the UPR^mt^ program. Of the 4610 significantly altered UPR^mt^ genes observed in WT cells, ~56% were under the regulatory control of CHOP, ATF4, or ATF5 (Fig. 6E). The 2568 genes regulated by CHOP, ATF4, and ATF5 displayed a mosaic pattern of regulation requiring either one, two, or all three transcription factors (Fig. 6, F and G), with a small subset of genes being regulated redundantly by the three transcription factors (Fig. 6G, highlighted red). A large set of genes (907) required all three transcription factors (Fig. 6F), followed by ATF4 alone (534), or a combination of CHOP and ATF5 (289). Of the three transcription factors, CHOP and ATF5 were the most related in terms of their UPR^mt^ signaling arms (Fig. 6G and fig. S7, A and B), whereas ATF4 had a more distinct UPR^mt^ signaling arm. Unique genetic footprints were also observed for each transcription factor (Fig. 6G, see dashed boxed regions), but the uniquely regulated genes were associated with GO categories common to all three transcription factors including regulation of cell cycle, response to oxidative stress, ubiquitin signaling and proteasomal degradation, and Wnt signaling. Some unique GO categories were also detected, including tRNA aminoacylation for ATF4, mRNA polyadenylation for ATF5, and O-glycan processing for CHOP (Fig. 6G). Genes associated with one carbon metabolism, which is remodeled in response to mitochondrial stress (*39*), were redundantly regulated by CHOP, ATF4, and ATF5.

**Fig. 7.**
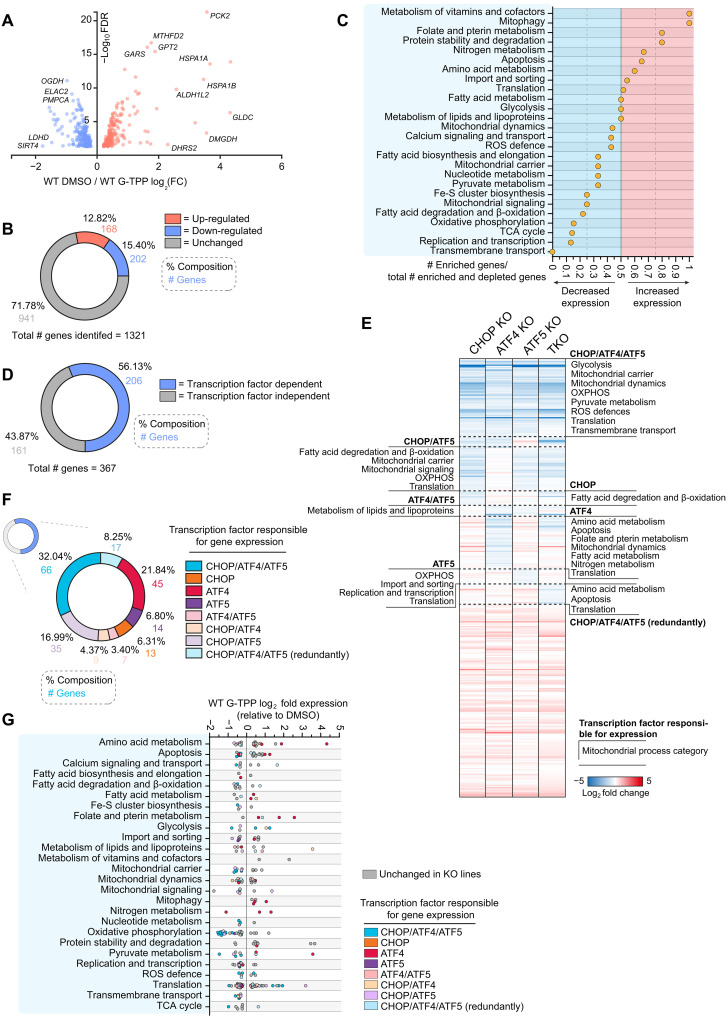
CHOP, ATF4, and ATF5 regulate mitochondrial gene expression through the UPR^mt^. (**A**) Graph of the expression changes of genes encoding mitochondrial proteins significantly altered in expression within the WT UPR^mt^ transcriptome. (**B**) Pie chart breakdown of the percentage and number of mitochondrial genes identified in the WT transcriptome that are altered or unchanged with UPR^mt^ induction. (**C**) Mitochondrial genes significantly altered in expression with UPR^mt^ induction were grouped by mitochondrial process and the relative number of genes in each process category showing increased expression with UPR^mt^ induction were graphed. (**D**) Pie chart breakdown of mitochondrial UPR^mt^ transcriptome genes that were unchanged ("transcription factor independent") or significantly decreased in expression in CHOP KO, ATF4 KO, ATF5 KO or TKO cells treated with G-TPP for 12 hours ("transcription factor dependent"). (**E**) Heatmap analysis of gene expression trends across the mitochondrial UPR^mt^ transcriptome in CHOP KO, ATF4 KO, ATF5 KO, and TKO cells treated with G-TPP for 12 hours, calculated relative to gene expression trends of WT cells treated with G-TPP for 12 hours. Enriched mitochondrial process categories identified in regulatory subgroups are labeled along the corresponding section of the heatmap. (**F**) Compositional breakdown displayed by pie chart of the overlapping regulatory patterns of CHOP, ATF4, and ATF5 in the transcription factor–dependent portion of the mitochondrial UPR^mt^ transcriptome. (**G**) Mitochondrial UPR^mt^ transcriptome trends in WT cells treated with G-TPP for 12 hours have been separated by mitochondrial process grouping and graphed. Genes have been labeled according to the transcription factor KO lines that showed decreased gene expression relative to WT in 12-hour G-TPP–treated samples. Data in (A) to (G) represent mean data calculated from three independent experiments.

The global transcriptome analysis in Fig. 6 can mask mitochondria associated genes since they represent only a small fraction of the total gene pool. To identify UPR^mt^-mediated mitochondrial changes that may have been masked in global gene analyses, a separate ontology analysis was performed on a curated gene set focused on mitochondria (*30*). Approximately 28% (370 of 1321) of the mitochondria associated gene set was altered in expression following UPR^mt^ activation (Fig. 7, A and B), and included up-regulation of genes related to protein stability and degradation, protein import/sorting, and metabolism of amino acids, fatty acids, folate, vitamins, and nitrogen (Fig. 7C). In contrast, genes involved in OXPHOS, the tricarboxylic acid (TCA) cycle, and transcription/translation-related processes were largely down-regulated by the UPR^mt^ (Fig. 7C).

Of the 367 UPR^mt^-regulated mitochondrial genes, ~56% were regulated by CHOP, ATF4, and ATF5 (Fig. 7D), and were associated with mitochondrial processes such as OXPHOS, reactive oxygen species (ROS) defense, import, and translation (Fig. 7E), and included mitochondrial DNA (mtDNA)–encoded genes (fig. S7D). The proportion of mitochondria associated genes regulated by one, two, or all three of CHOP, ATF4, and ATF5 (Fig. 7F), mirrored the observations made in the global gene analysis described above (Fig. 6F). Uniquely regulated processes were also identified and included import and sorting for ATF5, and folate and pterin metabolism for ATF4 (Fig. 7E). Notably, a portion of CHOP, ATF4, and ATF5 transcription factor activity involved maintaining the expression level of genes that were down-regulated by the UPR^mt^ (Fig. 7G). That is, genes that were down-regulated by the UPR^mt^, were down-regulated even further upon the loss of CHOP, ATF4, or ATF5. This occurred most strikingly for the OXPHOS gene subgroup, in which all OXPHOS genes regulated by either CHOP, ATF4, or ATF5 were further decreased in expression in transcription factor KO cells (see “Oxidative phosphorylation” in Fig. 7G). The three transcription factors therefore play a role in maintaining a certain level of privileged gene expression for the OXPHOS machinery that may help aid proteostasis recovery by fine-tuning protein levels. For example, fine-tuning of OXPHOS protein levels has been reported in *C. elegans* in which ATFS-1 down-regulated OXPHOS genes (*17*, *40*). However, in the mammalian system, it seems that there is a combined repression and activation of OXPHOS genes to fine-tune expression. Overall, the transcriptome analyses reveal that the transcriptional program of the UPR^mt^ drives various cellular and mitochondrial processes, in which CHOP, ATF4, and ATF5 drive distinct clusters of genes that function in common processes. The analysis demonstrates that CHOP, ATF4, and ATF5 work in concert during the UPR^mt^, with the signaling arms driven by CHOP and ATF5 being related to each other, whereas ATF4’s signaling arm is quite distinct.

We noted that CHOP, ATF4, and ATF5 were dispensable for ~44% of the global UPR^mt^ program (Fig. 6E), indicating that additional UPR^mt^ transcription factors remain to be identified. The RegNetwork database was used to identify common promotor and expression patterns throughout the gene set (fig. S7C) (*41*). The analysis produced a list of 30 putative UPR^mt^ genes including *MYC* and *MAX* that have previously been shown to function in chromatin modification (*42*), *YY1* that is known to affect mitochondrial related gene transcription (*43*, *44*), and *EP300* that has recently been identified to function during UPR^mt^ signaling (*45*) (fig. S7C). These transcription factors represent interesting candidates for future analysis.

### The UPR^mt^ sustains PINK1/Parkin mitophagy to promote mitochondrial recovery from stress

**Fig. 8.**
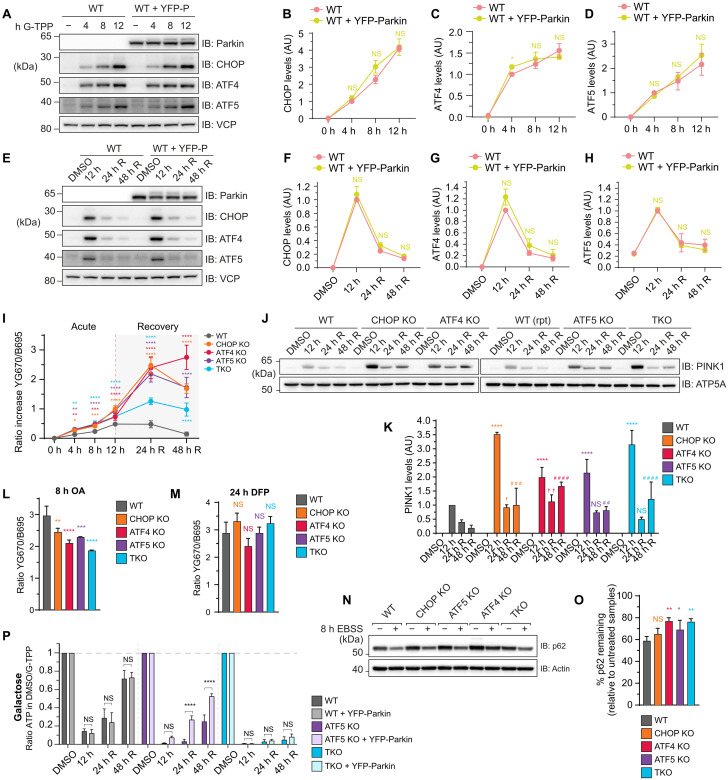
A unidirectional signaling relationship promotes PINK1/Parkin mitophagy activation during UPR^mt^-mediated recovery from proteostasis stress. (**A** to **H**) WT cells with and without YFP-Parkin (YFP-P) expression underwent the indicated G-TPP treatments and recovery time course and transcription factor expression was analyzed by immunoblot [(A) and (E)] and quantified relative to WT 4-hour [(B) to (D)] or WT 12-hour G-TPP sample expression [(F) to (H)]. (**I**) Indicated cell lines expressing YFP-Parkin and mtKeima underwent acute or recovery G-TPP treatment time courses and were analyzed for lysosomal-positive mtKeima using fluorescence-activated cell sorting (FACS). (**J** and **K**) PINK1 levels in mitochondria isolated from indicated cell lines at the labeled time points were analyzed by immunoblot (J) and quantified relative to WT 12-hour PINK1 expression (K). (**L** and **M**) Indicated cell lines expressing YFP-Parkin and mtKeima were treated with oligomycin/antimycin A (OA) (L) or deferiprone (DFP) (M) and analyzed for lysosomal-positive mtKeima by FACS. (**N** and **O**) Indicated cell lines were incubated in standard medium (−) or Earle’s balanced salt solution (EBSS) for 8 hours, and levels of p62 were analyzed by immunoblot (N) and quantified relative to standard medium treated samples (O). (**P**) Cellular ATP levels in WT, ATF5 KO, and TKO cells with and without YFP-Parkin expression in galactose-based medium were analyzed at the indicated time points and the ratio of ATP relative to DMSO-treated samples was calculated and graphed. Data in (B) to (D), (F) to (H), (I), (K) to (M), (O), and (P) represent mean ± SD from three independent experiments. *P < 0.05, **P < 0.005, ***P < 0.001, and ****P < 0.0001, [(B) to (D), (F) to (H), (I), and (P)] two-way ANOVA, relative to WT; [(L) and (M)] one-way ANOVA, relative to WT; (K) two-way ANOVA, relative to WT: * = 12 hours, † = 24-hour R, # = 48-hour R). Ratio increase in lysosomal-positive mtKeima was calculated relative to DMSO-treated samples [(I), (L), and (M)] (*47*). R, recovery.

PINK1/Parkin mitophagy and the UPR^mt^ are both activated in response to mitochondrial protein folding stress (*10*, *11*). To address whether there is interplay between the quality control pathways, we assessed whether PINK1/Parkin mitophagy and the UPR^mt^ influence the activity of one another. CHOP, ATF4, and ATF5 induction was analyzed by immunoblotting in response to G-TPP treatment in WT cells with and without Parkin expression (Fig. 8, A to H). No significant difference in CHOP, ATF4, or ATF5 levels was observed during acute proteostatic damage (Fig. 8, A to D) or during recovery time points (Fig. 8, E to H), indicating that PINK1/Parkin mitophagy does not affect UPR^mt^ signaling.

Next, the influence of UPR^mt^ signaling on PINK1/Parkin mitophagy activity was assessed using the mitochondrial-targeted fluorescent reporter Keima (mtKeima) (*5*, *46*, *47*). In WT cells, PINK1/Parkin mitophagy levels were highest at 12 hours of G-TPP treatment and persisted at 24 hours of recovery before returning to baseline by 48 hours of recovery (Fig. 8I). All UPR^mt^-deficient KO lines had slightly higher levels of mitophagy and followed a similar pattern to WT cells during the acute stress time points (4- to 12-hour G-TPP). However, during the 24- to 48-hour recovery time points, there was a large spike in mitophagy levels in CHOP, ATF4, and ATF5 KO lines (Fig. 8I), demonstrating that PINK1/Parkin mitophagy is hyperactivated when the UPR^mt^ is defective. Analysis of yellow fluorescent protein (YFP)–Parkin translocation to mitochondria following 12 hours of G-TPP treatment in WT cells showed a piecemeal type of mitophagy in which portions of the mitochondrial network were targeted by YFP-Parkin (fig. S8A), as opposed to the robust translocation observed following mitochondrial depolarization (fig. S8B). The piecemeal morphology is consistent with prior observations of Parkin translocation in cells ectopically expressing a misfolded form of the mitochondrial protein ornithine transcarbamylase (*48*). Higher levels of YFP-Parkin translocation to mitochondria were observed in ATF5 KO cells in a piecemeal fashion (fig. S8A), consistent with the elevated mitophagy levels observed in the mtKeima analyses (Fig. 8I).

Interestingly, mtKeima analysis of TKO cells showed that they did not have the same high increase in mitophagy levels as the single transcription factor KO cells (Fig. 8I). Instead, only a comparatively mild elevation in mitophagy was observed during the recovery period despite TKOs having an equally or more severe proteostasis stress relative to single KOs (Figs. 2 to 4). We assessed PINK1 accumulation (Fig. 8, J and K) and Parkin translocation (fig. S8A), and depolarization-induced PINK1/Parkin mitophagy (Fig. 8L and fig. S8B) but did not observe any significant defects in either measure in TKO cells relative to single KOs. Receptor-mediated mitophagy that is independent of PINK1/Parkin (Fig. 8M), and starvation-induced autophagy (Fig. 8, N and O), also did not show a significant defect in TKO cells relative to single KO controls, ruling out a generalized autophagy defect in the TKO line. The lowered levels of PINK1/Parkin mitophagy in TKO cells therefore appears to be a specific defect in sustaining prolonged PINK1/Parkin mitophagy activity in response to proteostasis stress (Fig. 8I).

To investigate whether PINK1/Parkin mitophagy functions alongside the UPR^mt^ to protect and repair mitochondrial dysfunction, OXPHOS derived cellular ATP levels were measured in WT, ATF5 KO, and TKO cells with and without Parkin expression. In WT cells, PINK1/Parkin mitophagy had no effect on ATP levels across the acute stress and recovery time course (Fig. 8P). In contrast, cellular ATP levels recovered significantly faster during the recovery period in ATF5 KO cells in the presence of PINK1/Parkin mitophagy (Fig. 8P). TKO cells failed to recover their ATP levels even in the presence of Parkin expression (Fig. 8P), consistent with their inability to drive sustained mitophagy (Fig. 8I). These results demonstrate that the UPR^mt^ alone is sufficient to repair OXPHOS function during protein folding stress, but when the UPR^mt^ is defective or perhaps overwhelmed, PINK1/Parkin mitophagy responds with elevated levels that are sustained redundantly by CHOP, ATF4, and ATF5 to facilitate OXPHOS recovery. Therefore, there appears to be a unidirectional communication between the quality control pathways in which PINK1/Parkin mitophagy is influenced by the activity of the UPR^mt^ but not the other way around.

## DISCUSSION

Stressed proteomes undergo widespread remodeling that goes beyond changes in individual protein levels, including protein misfolding. Given this, it is important to capture functional changes in proteomes to better understand how cells respond to stress (*49*–*52*). In MitoPQ, we have developed a functional proteomics framework that is specialized for the analysis of mitochondrial proteostasis. MitoPQ combined with KO lines of CHOP, ATF4, and ATF5 enabled us to address some of the fundamental roles of the UPR^mt^ in maintaining mitochondrial proteostasis during protein folding stress. Our analyses show that the UPR^mt^ functions across two distinct phases of protein folding stress, beginning with a protection phase that maintains proteostasis during an insult, and then a repair phase that restores proteostasis following recovery from an insult. The role of the UPR^mt^ during the protection phase was surprising given that inhibition of protein import is thought to be a signature of mitochondrial protein folding stress (*53*). How might the UPR^mt^ serve to protect proteostasis if the import of protective factors into mitochondria is inhibited? The answer lies in recent work revealing that changes in protein import during proteostatic stress occur temporally. It begins with a UPR^mt^-mediated boost in import early during the stress (*54*, *55*), followed by decreased protein import as the stress becomes more severe (*55*, *56*). Therefore, CHOP, ATF4, and ATF5 enact their UPR^mt^ program of proteostasis protection early during G-TPP treatment, before the more severe collapse of the import machinery that likely occurs by the end of the 12-hour treatment time point. However, during recovery from the insult when G-TPP is removed, it is likely that mitochondrial protein import is re-established to enable the UPR^mt^ to function in the second phase of the UPR^mt^ involving proteostasis repair.

Mitochondria are central hubs of metabolism that generate ATP through OXPHOS. MitoPQ analyses reveal that UPR^mt^ activity is highly focused on maintaining OXPHOS metabolism during proteostasis stress (Figs. 3, 4, and 8). The UPR^mt^ does so directly by focusing on the protection and repair of complex I of the OXPHOS machinery, and indirectly by protecting and repairing processes that support OXPHOS metabolism, including cardiolipin biosynthesis that supports OXPHOS supercomplexes and their activity (*57*, *58*), fatty acid metabolism that provides NADH, and ubiquinone biosynthesis that provides electron carriers. The mito-ribosome was also found to be highly reliant on the UPR^mt^ (Fig. 3), and it is largely dedicated to the production of mtDNA-encoded OXPHOS proteins, the majority of which are complex I subunits (*59*). The UPR^mt^ program mediated by CHOP, ATF4, and ATF5 was additionally identified to finely tune levels of OXPHOS transcripts (Fig. 7), likely to aid with syncing protein production with the protein folding capacity of mitochondria during stress. In addition to protecting and repairing OXPHOS metabolism, the UPR^mt^ through CHOP, ATF4, and ATF5 was required for elevated levels of transcripts related to one‑carbon metabolism and fibroblast growth factor 21 (FGF21) induction (Fig. 6G and table S9), both of which are involved in metabolic rewiring during mitochondrial stress (*60*). Given that deficiencies in complex I activity have been associated with Parkinson’s disease (*61*, *62*), it would be interesting to determine whether the UPR^mt^ contributes to preventing the progression of Parkinson’s disease pathogenesis via maintaining OXPHOS metabolism. Indeed, there are links for the UPR^mt^ in protecting dopaminergic neurons in *C. elegans* defective in PINK1/Parkin mitophagy (*63*), while the UPR^mt^ has also been linked to Alzheimer’s disease (*64*).

It is noteworthy to highlight that proteostasis disruption of the matrix compartment resulted in stress also occurring in the IMS when the UPR^mt^ was dysfunctional (Fig. 4, H and I). However, in contrast to the role of UPR^mt^ in both protecting and repairing metabolic hubs in the matrix (Figs. 3 and 4), the UPR^mt^ was only relied upon for protection, but not repair of the IMS compartment. It is therefore likely that the IMS has its own stress response program that drives repair of the compartment (*65*). In contrast, mitochondrial networking had a higher reliance on the UPR^mt^ during the repair phase as opposed to the protection phase (Fig. 5).

Through MitoPQ analyses, we found that CHOP, ATF4, and ATF5 were equally important for protecting and repairing proteostasis (Figs. 2 and 3), leading to the question of whether they drive a singular UPR^mt^ program or whether they each govern distinct nodes of the UPR^mt^ program. Overall, the transcriptome analyses showed that CHOP, ATF4, and ATF5 act in concert with each other by driving broadly overlapping gene sets, but with each transcription factor also controlling distinct gene sets that function in common pathways. In addition, CHOP, ATF4, and ATF5 were induced largely independently of one another (Fig. 2, A to C), further supporting the conclusion that they drive independent arms of the UPR^mt^ that function in concert. The requirement for multiple transcription factors to drive the UPR^mt^ in mammals is consistent with the UPR^mt^ in *C. elegans* that is governed by ATFS-1 and DVE-1 (*14*, *16*). However, it is likely that additional transcription factors of the mammalian UPR^mt^ remain to be identified since our transcriptome analyses revealed that CHOP, ATF4, and ATF5 drive only ~50% of the total UPR^mt^ program (Fig. 6). Multiple putative transcription factors and coregulators may be driving the remainder of the UPR^mt^ program (fig. S7C), including the histone acetyltransferase EP300 that has recently been linked to UPR^mt^ signaling in *C. elegans* and mammals (*45*). In addition, large transcriptome nodes related to the regulation of histone methylation were identified in the UPR^mt^ program that were not entirely under the control of CHOP, ATF4, and ATF5 (Fig. 6, C and G), indicating that the unidentified signaling lineages may require epigenetic remodeling reminiscent of DVE-1/lin-65 mediated chromatin remodeling arm in the *C. elegans* UPR^mt^ (*16*).

PINK1/Parkin mitophagy has been observed to activate in response to proteostasis stresses that also activate the UPR^mt^ (*10*, *11*, *56*, *66*). However, the interplay between these pathways has been largely unclear in mammalian systems. We identified unidirectional signaling between the two quality control pathways in which PINK1/Parkin mitophagy was influenced by the UPR^mt^ but not the other way around (Fig. 8). For example, PINK1/Parkin mitophagy was highly elevated in the absence of a functional UPR^mt^ (Fig. 8I), but UPR^mt^ signaling was similar in the presence or absence of PINK1/Parkin mitophagy (Fig. 8, A to H). Our results are consistent with a model in which PINK1/Parkin mitophagy functions as a last resort quality control mechanism that disposes of mitochondria that are damaged beyond the repair capacity of the UPR^mt^. The activity of PINK1/Parkin mitophagy under conditions where the UPR^mt^ was unable to restore proteostasis is important for maintaining OXPHOS metabolism (Fig. 8P), and likely also helps to prevent highly damaged mitochondria from herniating and releasing inflammatory mtDNA (*67*–*69*). Importantly, CHOP, ATF4, and ATF5, were found to redundantly sustain high levels of PINK1/Parkin mitophagy under conditions of severe proteostasis damage during the repair phase (Fig. 8I), demonstrating a role for the UPR^mt^ in supporting mitophagy.

In conclusion, through the development of a functional proteomics framework in MitoPQ, we define fundamental roles for the UPR^mt^ in protecting and repairing mitochondrial proteostasis, in which OXPHOS metabolism is a key UPR^mt^ target. The transcription factors CHOP, ATF4, and ATF5 are important UPR^mt^ players that function in concert to drive ~50% of the UPR^mt^ program that protects and repairs proteostasis, while unidirectional interplay between the UPR^mt^ and PINK1/Parkin mitophagy maintains OXPHOS activity when the UPR^mt^ is overwhelmed or dysfunctional.

### Limitations

In this study, we define the role of the UPR^mt^ following proteostasis stress generated through chaperone inhibition. It would be valuable in the future to also assess proteostasis stress alternatives, for example, the use of LONP1 inhibitor (CDDO), or ectopic expression of misfolded proteins in mitochondria (*9*, *10*, *26*, *70*). The large proteomic and transcriptomic datasets in this study represent a valuable resource for further exploration of the signaling and mechanisms of the UPR^mt^, and while we provide biological validation of OXPHOS activity and the interplay of PINK1/Parkin mitophagy with the UPR^mt^ in maintaining OXPHOS proteostasis, further biological validation of the transcriptional pathways and their roles in regulating mitochondrial proteostasis would be beneficial. Such analyses will likely yield important mechanistic insights into the UPR^mt^. Finally, our work showing that each transcription factor is a key player in proteostasis demonstrates their individual importance, but their tissue specificity remains to be determined and is an interesting area to explore.

## MATERIALS AND METHODS

### Experimental model and subject details

All HeLa (American Type Culture Collection, CCL-2.2) and human embryonic kidney 293T (CRL-3216) cell lines in this study were cultured in Dulbecco’s modified Eagle’s medium supplemented with 10% (v/v) fetal bovine serum (FBS) (Cytiva), 1% penicillin-streptomycin, 10 mM Hepes, GlutaMAX (Life Technologies, catalog no. 35050061), and nonessential amino acids (Life Technologies).

### Method details

#### Transfection reagents and antibodies

Transfection reagents including Lipofectamine LTX (Life Technologies, catalog no. A12621) and X-tremeGENE9 (Roche, catalog no. 6365787001) were used according to the manufacturers’ instructions. All commercial antibodies used in this study are listed in table S5.

### Generation of KO lines using CRISPR-Cas9 and TALEN gene editing

CHOP KO clone #2 (RRID: CVCL_C8SW) cells (used in all experiments except where noted in fig. S2) were generated using a TALEN that targets an exon common to all splicing variants (listed table S3). The TALEN constructs were generated by sequential ligation of coding repeats into pcDNA3.1/Zeo-Talen(+63), as previously described (*71*). CHOP KO #15 (RRID: CVCL_C8SV), ATF4 KO (RRID: CVCL_C8ST), ATF5 KO (RRID: CVCL_C8SU), DELE1 KO (RRID: CVCL_C8SY), and CHOP/ATF4/ATF5 TKO (RRID: CVCL_C8SX) cells were generated using CRISPR guide RNAs that target a common exon of all splicing variants of each gene. Oligonucleotides (Sigma-Aldrich) that contain CRISPR sequences were annealed and ligated into Bbs I–linearized pSpCas9(BB)-2A-GFP vector (*72*) (a gift from F. Zhang; Addgene plasmid # 48138; RRID: Addgene_48138). Sequence-verified guide RNA constructs were then transfected into HeLa cells for 24 hours and green fluorescent protein (GFP)–positive cells were individually sorted by fluorescence-activated cell sorting (FACS) into 96-well plates. Single-cell colonies were screened for the loss of the targeted gene product by immunoblotting after treatment with 300 nM thapsigargin for 8 hours (CHOP, ATF4, and ATF5) or by three-primer polymerase chain reaction (PCR) screening for genomic edits (DELE1) (*73*). Attempts at sequencing ATF4 KO #40 did not produce readable sequencing data, so ATF4 KO #40 was confirmed by Western blot. The presence of frameshift indels in the genes of interest in KO clones from immunoblotting or PCR screening was confirmed by Sanger sequencing. Genomic DNA was first isolated, and PCR was performed to amplify the targeted regions. For CHOP KO and ATF5 KO cells, the PCR products were subsequently cloned into a pGEM4Z vector for sequencing analysis (see table S4 for genotyping primers). For ATF4 KO and DELE1 KO cells, the PCR products were directly sequenced using sequencing primers that anneal to the amplified regions (table S4). The sequencing data for the control and the KO cells were then analyzed using Synthego ICE v2 CRISPR Analysis Tool (https://synthego.com/products/bioinformatics/crispr-analysis) (table S3). CHOP/ATF4/ATF5 TKO cells were generated by sequential transfections of CHOP TALEN plasmid into WT HeLa cells, then ATF5 CRISPR plasmid into CHOP KO cells, and then ATF4 KO CRISPR plasmid into CHOP/ATF5 DKO cells. All cell line clones used in each experiment are as follows, unless otherwise specified in fig. S2: CHOP KO #2, ATF4 KO #17, ATF5 KO #17, DELE1 KO #16, and CHOP/ATF4/ATF5 TKO #1. The protocol for the generation and design of CRISPR target constructs can be found online at https://doi.org/10.17504/protocols.io.j8nlkkzo6l5r/v1. The protocol for sequencing CRISPR edits can be found online at https://doi.org/10.17504/protocols.io.8epv59yx5g1b/v1.

### Generation of stable cell lines

pBMN-YFP-Parkin (RRID: Addgene_59416) and pCHAC-mt-mKeima (RRID: Addgene_72342) plasmids were described previously (*5*, *8*). Retroviruses were assembled in human embryonic kidney 293T cells and purified using lentivirus precipitation solution (ALSTEM) as per the manufacturer’s instructions. Supernatants containing purified virus were applied onto HeLa cells for 48 hours in the presence of polybrene (8 μg/ml; Sigma-Aldrich). Following transduction, the cells were recovered in full growth medium for 5 to 7 days, and protein expression levels among cell lines were matched by fluorescence sorting via FACS. The detailed protocol can be found online at https://doi.org/10.17504/protocols.io.261ged38yv47/v1.

### Proteostasis stress, mitophagy, and starvation treatments

For proteostasis stress experiments, cells were fed in full growth medium for 1 hour before treatment with 9 μM G-TPP (Advanced Molecular Technologies, catalog no. AMTA073-GT18) in full growth medium for the indicated times. For proteostasis recovery experiments, after treatment with G-TPP for 12 hours, cells were washed three times in excess phosphate-buffered saline (PBS) and treatment medium was replaced with full growth medium. Growth medium was replaced with fresh growth medium after 24-hour recovery. The protocol for proteostasis stress induction is online at https://doi.org/10.17504/protocols.io.j8nlkowr1v5r/v1. For mitophagy experiments, cells were treated with 10 μM oligomycin (Calbiochem, catalog no. 495455), 4 μM antimycin A (Sigma-Aldrich, catalog no. A8674), and 10 μM Quinoline-Val-Asp-Difluorophenoxymethylketone (QVD) (MedChemExpress, catalog no. HY-12305) for oligomycin/antimycin A treatment or 1 mM deferiprone (DFP; Sigma-Aldrich, catalog no 379409) for DFP treatment for the indicated times. The protocol for oligomycin/antimycin A–mediated mitophagy induction can be found online at https://doi.org/10.17504/protocols.io.14egn32yql5d/v1. The protocol for DFP-mediated mitophagy induction can be found online at https://doi.org/10.17504/protocols.io.rm7vzxbqrgx1/v1. For starvation experiments, cells were fed in full medium for 1 hour before 8-hour starvation in Earle’s balanced salt solution (Life Technologies, catalog no. 24010-043). The protocol for starvation stress induction can be found online at https://doi.org/10.17504/protocols.io.5jyl8pjq7g2w/v1.

### Immunoblotting

Cells were lysed in 1× lithium dodecyl sulfate (LDS) sample buffer (Life Technologies, catalog no. NP0007) in the presence of 100 mM dithiothreitol (DTT; Astral Biosciences, CAS: 3483-12-3) and heated at 99°C with shaking for 10 min. Mitochondria were lysed in 1× SDS sample buffer [5% (w/v) SDS, 10% (v/v) glycerol, 100 mM DTT, and 50 mM tris-Cl (pH 6.8)] and heated at 99°C with shaking for 10 min. 15 or 20 μg of mitochondria or 70 μg of cellular protein was subjected to NuPAGE Novex 4 to 12% bis-tris gels (Life Technologies) according to the manufacturer’s instructions and electro-transferred to polyvinyl difluoride membranes before immunoblotting using indicated antibodies (see table S5 for the antibodies used in this study). The protocol for whole-cell lysate preparation can be found online at https://doi.org/10.17504/protocols.io.e6nvwdj4dlmk/v1. The protocol for mitochondrial lysate preparation can be found online at https://doi.org/10.17504/protocols.io.n2bvj38enlk5/v1. The protocol for immunoblotting can be found online at https://doi.org/10.17504/protocols.io.j8nlkowy1v5r/v1.

### Mitochondrial isolation

Cell pellets were collected by scraping into cold PBS and frozen at −80°C to increase cell lysis. Pellets were then thawed and resuspended in cold isolation solution [20 mM Hepes (pH 7.6), 220 mM mannitol, 70 mM sucrose, 1 mM EDTA, and 0.5 mM phenylmethylsulfonyl fluoride] and homogenized with 30 strokes in a Dounce homogenizer. Lysates were then centrifuged at 800*g* at 4°C for 5 min to pellet nuclei and unbroken cells. Supernatants were then centrifuged at 10,000*g* at 4°C for 10 min to pellet mitochondria. Pelleted mitochondria were then washed once through resuspension in fresh isolation buffer and centrifugation at 10,000*g* at 4°C for 10 min to re-pellet mitochondria. The supernatant was removed, and mitochondrial pellets were resuspended in mitochondrial storage buffer [10 mM Hepes (pH 7.6) and 0.5 M sucrose]. Protein concentration was estimated spectroscopically, and aliquots of mitochondria were stored at −80°C until use. The detailed protocol for mitochondrial isolation can be found online at https://doi.org/10.17504/protocols.io.5jyl8pjq7g2w/v1.

For oxygen consumption assays, cell pellets were collected by scraping cells into cold modified isolation buffer [70 mM sucrose, 210 mM mannitol, 1 mM EGTA, 0.5% (w/v) bovine serum albumin (fatty acid free), and 5 mM Hepes (pH 7.2)], and pellets were collected by centrifugation at 3000*g* for 5 min at 4°C. Mitochondria were isolated as above with the following minor modifications: Cell pellets were stored on ice before homogenization, and mitochondria samples were stored on ice and immediately assayed after quantification.

### Preparation of soluble and insoluble mitochondrial protein fractions for immunoblotting

Two aliquots of 15 μg of mitochondria (“total” sample and “fractionation” sample) were thawed on ice and pelleted by centrifugation at 10,000*g* for 5 min at 4°C. Mitochondria were then lysed on ice for 15 min in chilled lysis buffer [0.5% (v/v) Triton X-100 in PBS] at a ratio of 1 μl of lysis buffer: 1 μg of mitochondria. The total fraction sample was then set aside on ice, while the fractionation sample was centrifuged at 12,000*g* for 10 min at 4°C. Supernatants representing the soluble protein fraction were then gently removed by pipette and placed into a fresh microfuge tube that was then set aside on ice. The pelleted protein representing the insoluble protein fraction was washed by adding a volume of lysis buffer back to the microfuge tube that was equal to the volume and the soluble fraction that was removed, flicking each tube gently to wash the side of the tube, centrifuging each sample at 12,000*g* for 10 min at 4°C and removing the supernatant gently by pipette. A total of two washes were performed, and, after the final wash, an equal volume of lysis buffer to the soluble fraction initial removed was added back to each insoluble fraction.

Each total, soluble, and insoluble protein fraction for each experimental sample was warmed to room temperature, and 4× SDS lysis buffer was added to each sample at 1× [4× SDS lysis buffer: 20% (w/v) SDS, 400 mM DTT, 40% (v/v) glycerol, and 200 mM tris-Cl (pH 6.8)]. Each sample was boiled with shaking at 99°C for 10 min, cooled to room temperature, and sonicated for 2 min in a waterbath sonicator. Samples were then subjected to NuPage Novex 4 to 12% bis-tris gels (Life Technologies) according to the manufacturer’s instructions and electro-transferred to polyvinyl difluoride membranes before immunoblotting using indicated antibodies (table S5). The detailed protocol can be found online at https://doi.org/10.17504/protocols.io.q26g7py9qgwz/v1.

### Preparation of soluble and insoluble mitochondrial protein fractions for mass spectrometry

One aliquot of 80 μg of mitochondria (fractionation sample) for each experimental sample was thawed on ice. Soluble and insoluble protein fraction isolation was performed as described earlier (see the "Preparation of soluble and insoluble mitochondrial protein fractions for immunoblotting" section) up to the final addition of lysis buffer to the insoluble protein fraction. Following fractionation, 3 ng of recombinant diacylglycerol acyltransferase/mycolyltransferase (Ag85A) from *M. tuberculosis* (Abcam, ab124604) per 1 μg of starting mitochondrial sample was added to each fraction in a ratiometric manner. After equilibrating to room temperature, samples were solubilized by adding 2× SDS solubilization buffer to a final concentration of 1× [2× SDS solubilization buffer: 10% (w/v) SDS and 200 mM Hepes (pH 8.5)]. Each sample was sonicated in a waterbath sonicator for 10 min. TCEP (Pierce) to a final concentration of 10 mM and chloroacetamide (Sigma-Aldrich) to a final concentration of 40 mM were added to each lysate, and samples were incubated at 37°C standing for 45 min. Lysates were then acidified by adding phosphoric acid to a final concentration of 1.2% per sample. Binding buffer [100 mM triethylammonium bicarbonate (TEAB) (Sigma-Aldrich) and 90% (v/v) methanol (pH 7.1) with phosphoric acid (Sigma-Aldrich)] was added to each sample at a ratio of 1:7 sample volume to binding buffer. Samples were loaded onto S-Trap Mini columns (Protifi) and centrifuged at 6500*g* for 30 s, repeating until the total sample was loaded with the flow-through discarded between each spin. Columns were washed by adding 400 μl of binding buffer and centrifuging at 6500*g* for 30 s. Wash steps were repeated for a total of four washes. Following the final wash, columns were moved to LoBind microfuge tubes (Eppendorf) and 125 μl of digestion buffer (50 mM TEAB) supplemented with sequencing grade Trypsin (Promega) at a concentration of 1 μg of trypsin: 50 μg of equivalent starting sample protein was added directly to each column filter. Samples were centrifuged at 1000*g* for 30 s, and the digestion buffer flow-through was pipetted directly back onto each column filter. Columns in their collection tubes were then sealed with parafilm and incubated statically at 37°C for 16 hours. Digested peptides were then eluted from each column through adding to each sample 80 μl of digestion buffer (no trypsin) and centrifuging samples at 3200*g* for 60 s, adding 80 μl of 0.2% (v/v) formic acid (FA) and centrifuging samples at 3200*g* for 60 s, and adding 80 μl of 50% (v/v) acetonitrile/0.2% (v/v) FA and centrifuging samples at 6 500*g* for 60 s. Each sequential eluate was pooled together within each sample, and samples were lyophilized and stored at −80°C for downstream TMT labeling. The detailed protocol can be found online at https://doi.org/10.17504/protocols.io.kxygx394kg8j/v1.

### TMT labeling and reverse-phase high pH fractionation

Lyophilized peptide pellets were reconstituted in 100 mM TEAB, and concentration estimates were performed spectroscopically using a Nanodrop 1000 (Thermo Fisher Scientific). For each sample, 10 μg of peptides was aliquoted into a Lo-Bind microfuge tube (Eppendorf) and diluted to a total volume of 100 μl per sample in 100 mM TEAB. For the pooled batch control, 0.79 μg of each sample was combined in the same microfuge tube to bring the pooled batch control to a total of 100 μg of peptides and diluted to 100 μl of total volume in 100 mM TEAB. Ten-plex TMT labels to a final quantity of 1.6 mg per label were reconstituted in acetonitrile as per the manufacturer’s instructions, and 5.86 μl of the appropriate label was added to each sample according to the batch layout specified in table S8, while the total label volume was added to the batch control sample. Labeling reactions were performed as per the manufacturer’s instructions. The detailed protocol for TMT labeling can be found online at https://doi.org/10.17504/protocols.io.n92ldmp69l5b/v1. Labeled samples in each batch (table S8) were combined into a single tube, lyophilized, and stored at −80°C. Lyophilized samples were reconstituted in 300 μl of 0.1% (v/v) trifluoroacetic acid and loaded onto high pH reversed-phase fractionation columns (Pierce). Samples were fractionated as per the manufacturer’s instructions using a modified elution gradient (table S6). After elution, fractions were concatenated in an equidistant manner to generate six sample fractions per batch sample. Concatenated samples were lyophilized and stored at −80°C. The detailed protocol for peptide fractionation can be found online at https://doi.org/10.17504/protocols.io.yxmvm32bnl3p/v1.

### Liquid chromatography–mass spectrometry analysis

Using a Dionex UlitMate 3000 RSLCnano system equipped with a Dionex UltiMate 3000 RS autosampler, the samples were loaded via an Acclaim PepMap 100 trap column (100 μm.2 cm, nanoViper, C18, 5 μm, 100; Thermo Fisher Scientific) onto an Acclaim PepMap RSLC analytical column (75 μm.50 cm, nanoViper, C18, 2 μM, 100; Thermo Fisher Scientific). The peptides were separated by increasing concentrations of 80% (v/v) acetonitrile (ACN)/0.1% (v/v) formic acid (FA) at a flow of 250 nl/min−1 for 158 min and analyzed with an Orbitrap Fusion Tribrid mass spectrometer (Thermo Fisher Scientific) operated in data-dependent acquisition mode. Each cycle was set to a fixed cycle time of 2.5 s consisting of an Orbitrap full ms1 scan at a resolution of 120,000, a normalized AGC target of 50%, a maximum IT of 50 ms, and a scan range of 380 to 1580 mass/charge ratio (*m*/*z*). ms1 precursors were filtered by setting monoisotopic peak determination to peptide, intensity threshold to 5.0 × 10^3^, charge state to 2 to 6, and dynamic exclusion to 60 s. Precursors were isolated in the quadrupole with a 1.6-*m*/*z* isolation window, collected to a normalized AGC target of 40% or maximum injection time (150 ms), and then fragmented with a CID collision energy of 30%. For ms3 scans, spectra were then filtered with a precursor selection range of 400 to 1200 *m*/*z*, isobaric tag loss exclusion of TMT, and precursor mass exclusion set to 20 *m*/*z* low and 5 *m*/*z* high. Subsequently, 10 synchronous precursor ions were selected, and scans were acquired at a resolution of 50,000, a normalized AGC target of 100%, a maximum IT of 250 ms, and a scan range of 120 to 750 *m*/*z*.

### Calculation of mitochondrial protein solubility

Raw instrument files were processed using MaxQuant version 1.6.17 (RRID: SCR_014485) with the Andromeda search engine (*74*), searching against the UniProt human database containing reviewed and canonical isoform variants in the FASTA format (2021), with the recombinant Ag85A sequence added as custom entry in the human database. All raw data files were analyzed using the MaxQuant proteomics data analysis workflow using the Andromeda search engine with modifications (*75*). Briefly, liquid chromatography–mass spectrometry run was set to “Reporter ion MS,” and TMT11-plex labels were set as isobaric labels with a reporter ion mass tolerance of 0.003 Da. Trypsin/P cleavage specificity was used with a maximum of two missed cleavages. Oxidation of methionine and N-terminal acetylation were set as variable modifications, and carbamidomethylation of cysteine was set as a fixed modification. A search tolerance of 4.5 ppm was used for ms1, and 20 ppm was used for ms2 matching. False discovery rates (FDRs) were determined through the target-decoy approach and set to 1% for both peptides and proteins. The “Match between runs” option was enabled, with an FDR of 1% and a mass bin size of 0.0065 Da. Minimum unique and razor peptides was set to 1, and label minimum ratio count was set to 2. Data from the proteinGroups.txt output table was then normalized according to methods described previously (*76*). The internal spiked control (Ag85A) intensities were averaged across each reporter ion channel, and this value was used to generate scaling factors for each channel to normalize reporter ion intensities for each protein to the relative starting intensities in each sample at the time of Ag85A addition. After normalization, data were imported into Perseus v1.6.15 (RRID: SCR_015753) and “only identified by site,” “reverse,” and “potential contaminant” identifications were removed (*77*). For the TMT-labeled mass spectrometry data in Fig. 1, cleaned files from Perseus were imported into R (v4.0.3; RRID: SCR_001905) where the QRILC method of imputation was performed using the impute.LCMD package (v2.0; Lazar C, 2015; https://cran.rstudio.com/web/packages/imputeLCMD/index.html#:~:text=imputeLCMD%3A%20A%20Collection%20of%20Methods,non%2Dresponses%20in%20proteomics%20experiments) for proteins that were absent in one batch of either “soluble” or “insoluble” fraction groups (*78*). Cleaned data were then filtered according to a database of mitochondrial associated proteins to only include mitochondrial proteins for downstream analysis (*30*). Computationally derived total intensities were then generated for each protein in each sample through the addition of soluble and insoluble sample fraction intensities. A percentage of total protein that was in the insoluble fraction was then calculated using the computationally derived total intensity. AI analysis was performed by calculating the average percentage of protein that is insoluble across a defined process group of mitochondrial proteins. Rate recovery analysis was performed across the 48-hour recovery period for CHOP KO, ATF4 KO, ATF5 KO, and TKO cells and across the first 24-hour recovery period for WT cells as WT cells had already recovered to baseline solubility by 48-hour recovery. Solubility shifts were not calculated for proteins that were only detected in the soluble or insoluble protein fractions. The protocol for mitochondrial protein solubility calculation can be found online at https://doi.org/10.17504/protocols.io.81wgbxx7nlpk/v1.

### Mitophagy analysis using mtKeima

Cells stably expressing YFP-Parkin and mtKeima were seeded in 24-well plates 24 hours before treatment. Treatments as indicated by the appropriate figure legends were performed as described earlier (see the “Proteostasis stress, mitophagy, and starvation treatments” section). At the conclusion of the treatment time, cells were washed in 1× PBS, trypsinized, and then resuspended in ice-cold standard growth medium. Cell pellets were centrifuged at 1000*g* for 1.5 min, and the supernatant was removed by aspiration before resuspension in sorting medium [10% (v/v) FBS and 1 mM EDTA in PBS]. Sample suspensions were then analyzed using the FACSDiva software on a LSR Fortessa X-20 cell sorter (BD Biosciences). Lysosomal mtKeima was measured using dual excitation ratiometric pH measurements at 488-nm (pH 7) and 561-nm (pH 4) lasers with 695- and 670-nm detection filters, respectively. Additional channels used include GFP (Excitation/Emission, 488 nm/530 nm). Sample compensation was performed at the time of sample analysis using the FACSDiva software. A minimum of 30,000 events were collected per sample.

Data were processed using FlowJo v10.7.2 (RRID: SCR_008520). Samples were first gated to exclude debris and then gated for single cell events using the forward and side scatter measurements. Individual event emission values for each sample were exported, and mtKeima shifts were calculated according to published methods (*47*). The detailed protocol for mtKeima sample preparation can be found online at https://doi.org/10.17504/protocols.io.5qpvo3rd9v4o/v1.

### ATP measurements

Cells were seeded in white opaque 96-well plates (Corning) 24 hours before treatment. Samples assayed in glucose-based medium were treated with G-TPP as described earlier (see “Proteostasis stress, mitophagy and starvation treatments”). Samples assayed in galactose-based medium were placed into galactose medium [galactose medium: 25 mM d-galactose, 10% (v/v) dialyzed FBS (Gibco), 1% (v/v) penicillin-streptomycin, 10 mM Hepes (pH 7.2), GlutaMAX (Life Technologies), and nonessential amino acids (Life Technologies) in glucose-free Dulbecco’s modified Eagle’s medium (Life Technologies)] 12 hours before assay analysis (12-hour DMSO and 12-hour G-TPP) or 24 hours before assay analysis (24-hour R and 48-hour R). At each analysis time point (12-, 24-, and 48-hour R), ATP levels were assayed using the Mitochondrial ToxGlo Assay Kit (Promega) as per the manufacturer’s instructions on a FLUOstar OPTIMA (BMG LABTECH) plate reader. This protocol can be found online at https://doi.org/10.17504/protocols.io.dm6gp3jmjvzp/v1.

### Oxygen consumption assays

Isolated mitochondria were quantified by bicinchoninic acid assay (Pierce, catalog no. 23227) as per the manufacturer’s instructions. Aliquots of 15 μg of mitochondria were diluted to a final volume of 25 μl in mitochondrial assay solution [70 mM sucrose, 220 mM mannitol, 10 mM KH_2_PO_4_, 5 mM MgCl_2_·6H_2_O, 1 mM EGTA, 0.1% (w/v) bovine serum albumin (fatty acid free), and 2 mM Hepes (pH 7.2)] and left to rest on ice. An equilibrated Seahorse cartridge plate (Agilent) was loaded with 20 mM adenosine 5′-diphosphate, oligomycin (50 μg/μl), 10 μM carbonyl cyanide *p*-trifluoromethoxyphenylhydrazone, and 40 μM antimycin A, resulting in final sample plate concentrations of 2 mM, 5 μg/μl, 1 μM, and 4 μM, respectively. Cartridges were then incubated in a 37°C CO_2_-free incubator for 45 min. During this incubation, a 25-μl aliquot of mitochondria was added to each corresponding sample well of a prechilled sample plate on ice. Plates were immediately centrifuged at 2000*g* for 20 min at 4°C and left on ice until insertion into the Seahorse XFe96 Analyzer (Agilent) after the cartridge plate calibration cycle on the analyzer was complete. Immediately before insertion into the Seahorse XFe96 analyzer, 155 μl of prewarmed substrate solution (10 mM glutamate and 10 mM malate in mitochondrial assay solution buffer) was added to the corresponding sample wells. Oxygen consumption analysis cycles were performed sequentially as follows: basal (3 min mix, 3 min measure, 3 min mix, and 3 min measure), adenosine 5′-diphosphate (injection, 30 s mix, and 3 min measure), oligomycin (injection, 30 s mix, 30 s wait, and 3 min measure), carbonyl cyanide *p*-trifluoromethoxyphenylhydrazone (injection, 20 s mix, and 3 min measure), antimycin A (injection, 30 s mix, and 3 min measure). The protocol, from mitochondrial isolation to oxygen consumption analysis on the Seahorse analyzer, was repeated each day at the time of sample collection [12-hour G-TPP (day 1), 24-hour R (day 2), and 48-hour R (day 3)]. Time from isolation to assay start was consistent between all 3 days. DMSO-treated samples were analyzed alongside experimental samples at each time point. Raw data files were exported from Wave software (v2.6; https://www.agilent.com/en/product/cell-analysis/real-time-cell-metabolic-analysis/xf-software/seahorse-wave-desktop-software-740897) and imported into Microsoft Excel (RRID: SCR_016137). The average basal oxygen consumption of the DMSO-treated samples from the first time point (12-hour G-TPP) for each cell line was calculated and used to generate normalization factors to normalize variation in oxygen consumption measurements across each day of analysis. Normalized oxygen consumption rate values were then used to calculate the basal respiration rates and total respiratory capacity of each experimental sample. All data were then displayed using GraphPad Prism v9.1 (RRID: SCR_002798) and analyzed using the statistical methods listed in the corresponding figure legend. This protocol can be found online at https://doi.org/10.17504/protocols.io.3byl4qj6zvo5/v1.

### Quantitative reverse transcription PCR analysis

Total RNA was isolated using the Monarch Total RNA Miniprep Kit (New England Biolabs) as per the manufacturer’s instructions. Concentrations of RNA in each sample were determined spectroscopically, and cDNA libraries were synthesized using a High-Capacity cDNA Reverse Transcription Kit (Applied Biosystems) as per the manufacturer’s instructions, using oligo(dT)_20_ primers (Sigma-Aldrich). Libraries were diluted 1:4 in diethyl pyrocarbonate–treated H_2_O. Quantitative reverse transcription PCR (qRT-PCR) analysis was performed using QuantiNova SYBR Green PCR Master Mix (QIAGEN) as per the manufacturer’s instructions on a RotorGene Q (QIAGEN) PCR machine. Primers used in this study are located in table S7. Threshold values were set using Rotor-Gene Q Series software v2.3.5 (RRID: SCR_015740). Cycle threshold (Ct) values were used to calculated fold changes in mRNA levels using the 2^−ΔΔCt^ method (*79*). Transcription factor mRNA levels were normalized to glyceraldehyde-3-phosphate dehydrogenase (GAPDH) mRNA levels in each sample. The protocol for qRT-PCR sample preparation can be found online at https://doi.org/10.17504/protocols.io.4r3l22713l1y/v1.

### Immunofluorescence microscopy

For immunofluorescence microscopy, cells were cultured on HistoGrip (Thermo Fisher Scientific)–coated glass coverslips for 24 hours before experimental treatment. All steps were performed at room temperature. Samples were first fixed in 4% (w/v) paraformaldehyde in 0.1 M phosphate buffer (10 min), rinsed three times with PBS, permeabilized with 0.1% (v/v) Triton X-100 in PBS (10 min), and then blocked with 3% (v/v) goat serum in 0.1% (v/v) Triton X-100 in PBS (15 min). The samples were incubated with the indicated primary antibodies made up in 3% (v/v) goat serum in 0.1% (v/v) Triton X-100 in PBS for 2 hours, rinsed three times with PBS, and incubated with secondary antibodies conjugated to Alexa Fluor 488 and Alexa Fluor 555 (Thermo Fisher Scientific) made up in 3% (v/v) goat serum in 0.1% (v/v) Triton X-100 in PBS for 2 hours. Coverslips were rinsed three times with PBS and then mounted using a tris-buffered DABCO (1,4-diazabicyclo[2.2.2]octane)-glycerol mounting medium. The detailed protocol is available online at https://doi.org/10.17504/protocols.io.14egn32yyl5d/v1.

Imaging of samples for mitochondrial morphology and YFP-Parkin localization analyses were conducted on an inverted Leica Stellaris 8 confocal with FLIM under 40×/1.30 numerical aperture (oil immersion, HC PL APO, CS2, Leica Microsystems), using Leica Power HyD detector S and HyD detector X. All mitochondrial morphology images were acquired in three-dimensional (3D) by optical sectioning with a minimum z stack range of 2.1 μm and a maximum voxel size of 94.7 nm laterally (*x*, *y*) and 350 nm axially (*z*). All YFP-Parkin localization images were acquired in 3D by optical sectioning with a minimum z stack range of 3.6 μm and a maximum voxel size of 94.7 nm laterally (*x*, *y*) and 300 nm axially (*z*) using Leica Power HyD detector S and HyD detector X.

### Mitochondrial morphology analysis

For each experimental sample, five randomly selected fields of view were collected as described under the “Immunofluorescence microscopy” section totaling ~150 cells per sample. Channel signals for TOM20 and SDHA were combined in Fiji (v 2.9.0; http://fiji.sc/). Morphology was analyzed using the Mitochondrial Analyzer plugin in Fiji using standard parameters for 3D analysis, with the exception of the local threshold method that was changed to the median. Statistical analysis was then conducted using GraphPad Prism 9.

### RNA sequencing library preparation and analysis

RNA sequencing was performed with the support of Micromon Genomics (Monash University). Total RNA samples were extracted from cell pellets using an RNeasy Mini kit (QIAGEN) as per the manufacturer’s instructions. Total RNA was measured using a Qubit analyzer (Invitrogen) as per the manufacturer’s instructions, using 2 μl of each sample, and assayed with the Qubit RNA High Sensitivity Assay (Invitrogen) as per the manufacturer’s instructions. Each sample was sized and measured for RNA integrity using the Bioanalyzer 2100 (Agilent) and RNA Nano Assay kit (Agilent) as per the manufacturer’s instructions. Samples were then processed using an MGI RNA Directional Library Preparation Set V2 (polyadenylate), as per the manufacturer’s instructions with some modifications. Modifications were as follows: 500 ng of RNA was used as input, RNA was fragmented at 87°C for 6 min to target an insert size of 200 to 400 base pairs, adapters were diluted 1:5, and the libraries were amplified with 13 cycles of PCR. The libraries were then pooled in equimolar concentrations and sequenced using an MGI DNBSEQ-2000RS with reagent chemistry V3.1, aiming for ~30 million reads per sample. Bioinformatics analysis of NGS data was performed with the support of the Monash Bioinformatics Platform (Monash University). Analysis of sequencing reads was performed using the rnasik 1.5.4 pipeline with STAR aligner (RRID: SCR_004463; https://github.com/MonashBioinformaticsPlatform/RNAsik-pipe), using the GRCh38 (*Homo sapiens*) reference genome (*80*). A UPR^mt^-induced change in gene expression was defined as changes that were statistically significant (FDR ≤ 0.05) with increased or decreased expression relative to the WT DMSO control samples of >±0.2-fold. For mitochondrial encoded genes, sequenced reads were trimmed with Trim Galore (--fastqc --paired --nextera --clip_R1 1 --clip_R2 1) (https://github.com/FelixKrueger/TrimGalore; RRID: SCR_011847) using Cutadapt 1.18 (RRID: SCR_011841) (*81*) and FastQC 0.11.9 (https://github.com/s-andrews/FastQC; RRID: SCR_014583). Trimmed reads were quantified with Salmon 1.5.2 (RRID: SCR_017036) using the selective alignment procedure (-l A --seqBias --qcBias --validateMappings) against the GENCODE vM27 transcriptome, with custom mitochondrial transcripts (properly merged bicistronic transcript sequences and corrected terminal sequences and removal of mt-tRNAs). Transcript quantifications were summarized to gene level with tximport (RRID: SCR_016752) (*82*) and analyzed for differential gene expression changes with DESeq2 (RRID: SCR_015687) (*83*), using the apeglm (*84*) shrinkage estimator, and an lfcThreshold of log 2 (1.5). Gene expression changes with an FSOS s-value &lt; 0.01 were considered significant. Normalized, strand-specific coverage profiles were generated with deepTools 3.5.0 (RRID: SCR_016366) bamCoverage (--filterRNAstrand [forward/reverse] --samFlagInclude 2 --samFlagExclude 256 --normalizeUsing CPM --exactScaling -bs 1 -of bigwig).

### GO analysis

Sets of genes of interest were analyzed using the Database for Annotation, Visualization, and Integrated Discovery (DAVID) online bioinformatics tool (RRID: SCR_001881) for enriched clusters of Biological Process (GOTERM_BP_DIRECT) and Molecular Function (GOTERM_MF_DIRECT) categories (*85*). The largest GO term of each cluster was used as the representative GO cluster term. Cellular pathway enrichment analysis was performed using the KEGG database through the ShinyGO V0.75 (RRID: SCR_019213) bioinformatic enrichment tool (0.05 FDR cutoff) (*86*). Mitochondrial process group analysis was performed using a curated dataset of mitochondrial related proteins (*30*). Representative mitochondrial process group values were calculated by taking the average aggregation value of all detected proteins belonging to each process group in each sample.

### Data analysis

No statistical methods were used to predetermine sample size. For Western blots, band intensities were measured with ImageLab 6.1.0 (Bio-Rad; RRID: SCR_014210). All statistical analyses were performed using GraphPad Prism 9. Statistical significance was calculated from three independent experiments using one-way or two-way analysis of variance (ANOVA) (considered significant for *P* ≤0.05), as specified in the relevant figure legend. Error bars are reported as mean ± SD.
