# Supplementary material for: Temporal landscape of mitochondrial proteostasis governed by the UPRmt
Source: Sci Adv. 2023 Sep 22;9(38):eadh8228. doi: 10.1126/sciadv.adh8228 (PMC10516501; doi:10.1126/sciadv.adh8228)
Supplement: Supplementary file 1 — Figs. S1 to S8 Tables S1 to S7 Legends for tables S8 and S9 [file sciadv.adh8228_sm.pdf]

Science Advances
AAAS

Supplementary Materials for

Temporal landscape of mitochondrial proteostasis governed by the UPR^{mt}

Louise Uoselis *et al.*

Corresponding author: Michael Lazarou, lazarou.m@wehi.edu.au

Sci. Adv. **9**, eadh8228 (2023)
DOI: 10.1126/sciadv.adh8228

The PDF file includes:

Figs. S1 to S8
Tables S1 to S7
Legends for tables S8 and S9

Other Supplementary Materials for this manuscript includes the following:

Tables S8 and S9

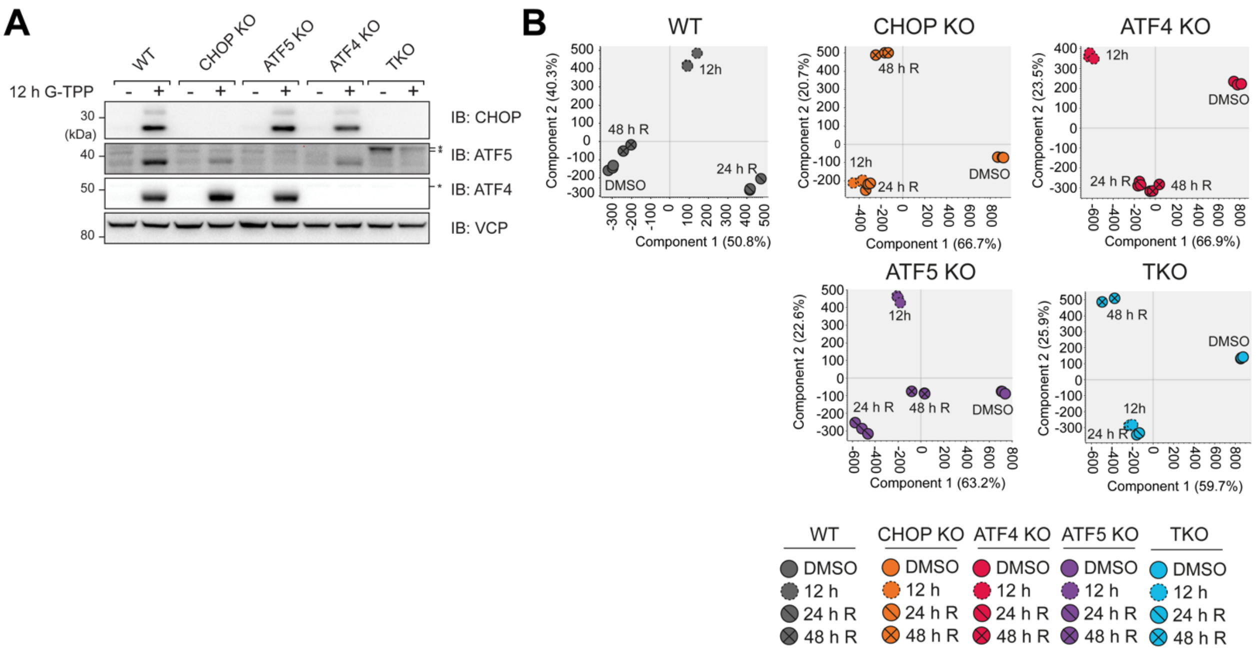

C

WT		CHOP KO		ATF4 KO		ATF5 KO		TKO	
COMPONENT 1	COMPONENT 2	COMPONENT 1	COMPONENT 2	COMPONENT 1	COMPONENT 2	COMPONENT 1	COMPONENT 2	COMPONENT 1	COMPONENT 2
MRPL54	TRUB2	SLC30A9	KIAA0141	CTSB	LIG3	LIG3	SYNJ2BP	GATB	SIRT3
PARS2	COQ7	LCLAT1	MRPL34	CYB5A	MDH1	MMS19	MRPL42	BNIP1	FTSJ2
MRPL42	CRAT	NME1	C12orf65	PGLS	CKMT1A	NUDT19	NUDT9	PACSIN2	COA1
MRPL30	CCDC109B	TOMM7	MT-CO1	C12orf65	TALDO1	PNPLA8	AGMAT	COX18	COX19
NUDT9	GRPEL2	MCL1	TMEM126B	STARD7	ATP5SL	AKAP1	MT-ATP6	REXO2	NDUFAF6
SLC25A35	FASTKD5	COX7A2L	COX19	GSTO1	SLC27A3	ARF5	C8orf82	NDUFB1	PRELID1
BCL2L1	ADCK4	IMMP1L	SLC25A44	LONP2	NDUFB1	TBC1D15	TUBB3	CARKD	PISD
ACADS	AARS2	HAGH	HIGD1A	GUK1	PTCD2	GCLM	BID	UQCC3	PDSS2
TIMM9	EARS2	TTC19	DHTKD1	SFXN2	ITPR2	ATP5SL	SPG7	OXSM	COQ7
MUL1	MTIF2	TOMM40L	QDPR	SLC25A28	IDE	STARD7	PDF	IMMP1L	ACAD11

Fig S1. Validation of cell lines and temporal MitoPQ analysis sample trends, related to Fig 2. (**A**) WT, CHOP KO, ATF4 KO, ATF5 KO and TKO cells were treated with G-TPP for 12 h and gene expression loss in each KO cell line was validated by immunoblot. (**B**) Principal component analysis of MitoPQ solubility values in each temporal data set, analyzed by each cell line. (**C**) The top 10 protein drivers from each component generated from the PCA plots in (B) for each cell line have been listed. Each data point in (B) represents one independent experimental sample.

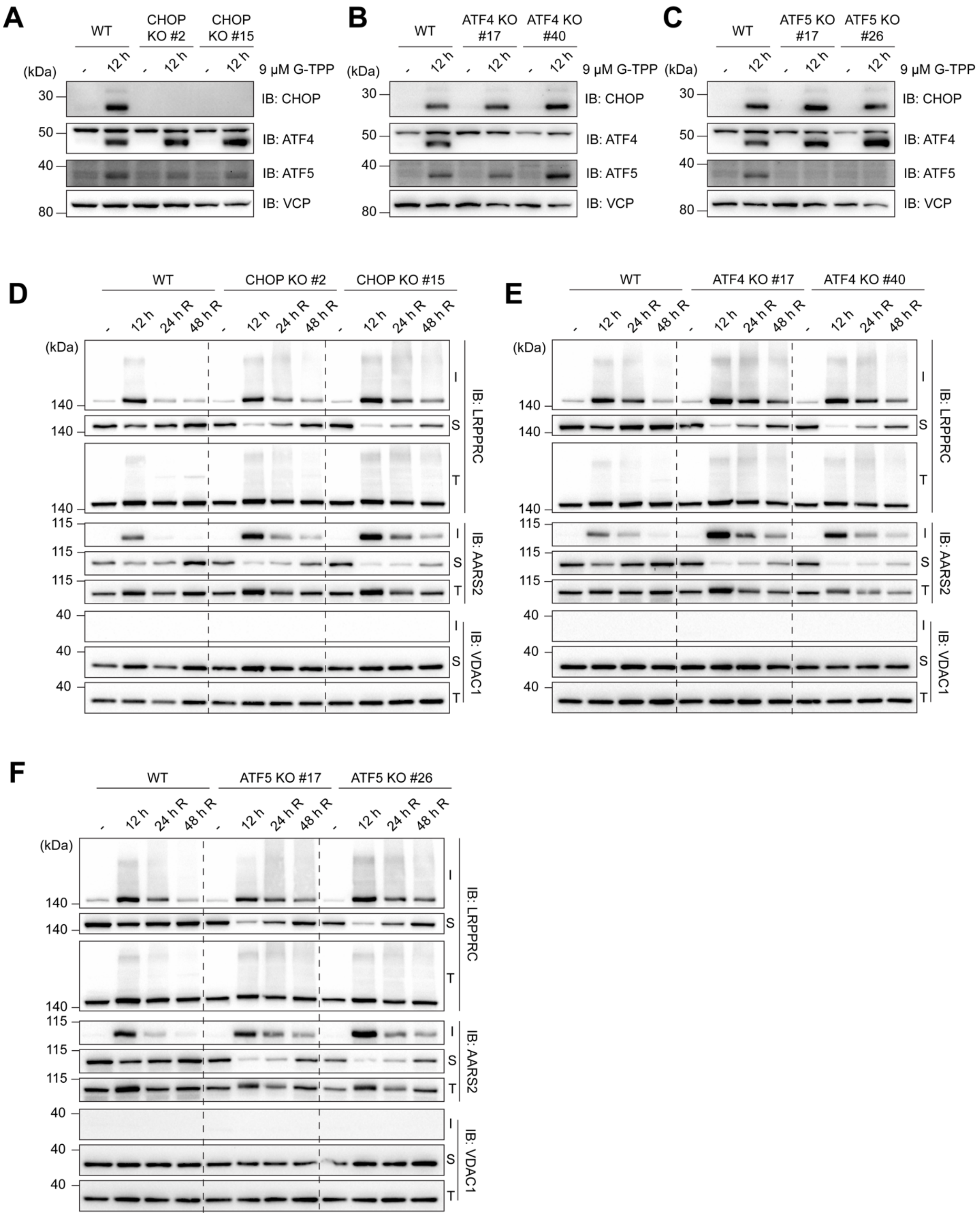

Fig S2. Validation of protein aggregation in additional KO clones, related to Figure 2. (**A-F**) WT, CHOP KO, ATF4 KO and ATF5 KO cells were treated with G-TPP for 12 h (**A-C**) with a 48 h recovery washout period. Transcription factor levels were analysed by immunoblot (IB) to

confirm a loss of protein expression in KO cells (**A – C**). (**D – C**) IB validation of protein solubility trends identified in Figure 2.

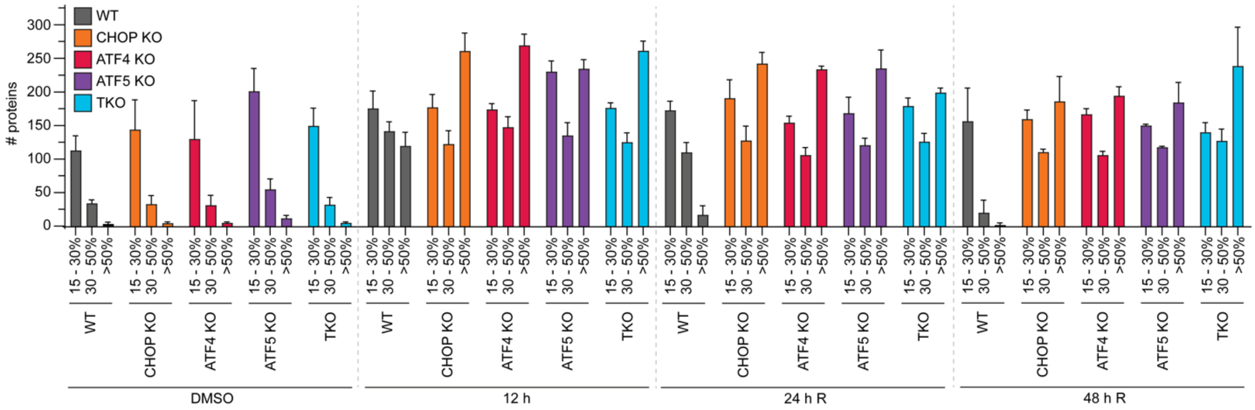

Fig S3. Elevated protein aggregation observed in both WT and UPR^{mt}-deficient cells is largely comprised of select, strongly aggregating proteins, related to Figs 2 and 3. Protein aggregation trends across the mitoproteome of WT, CHOP KO, ATF4 KO, ATF5 KO and TKO cells at the indicated time points were sub-grouped into the labelled % aggregation groupings and graphed. Data represents mean data ± SD from three independent experiments.

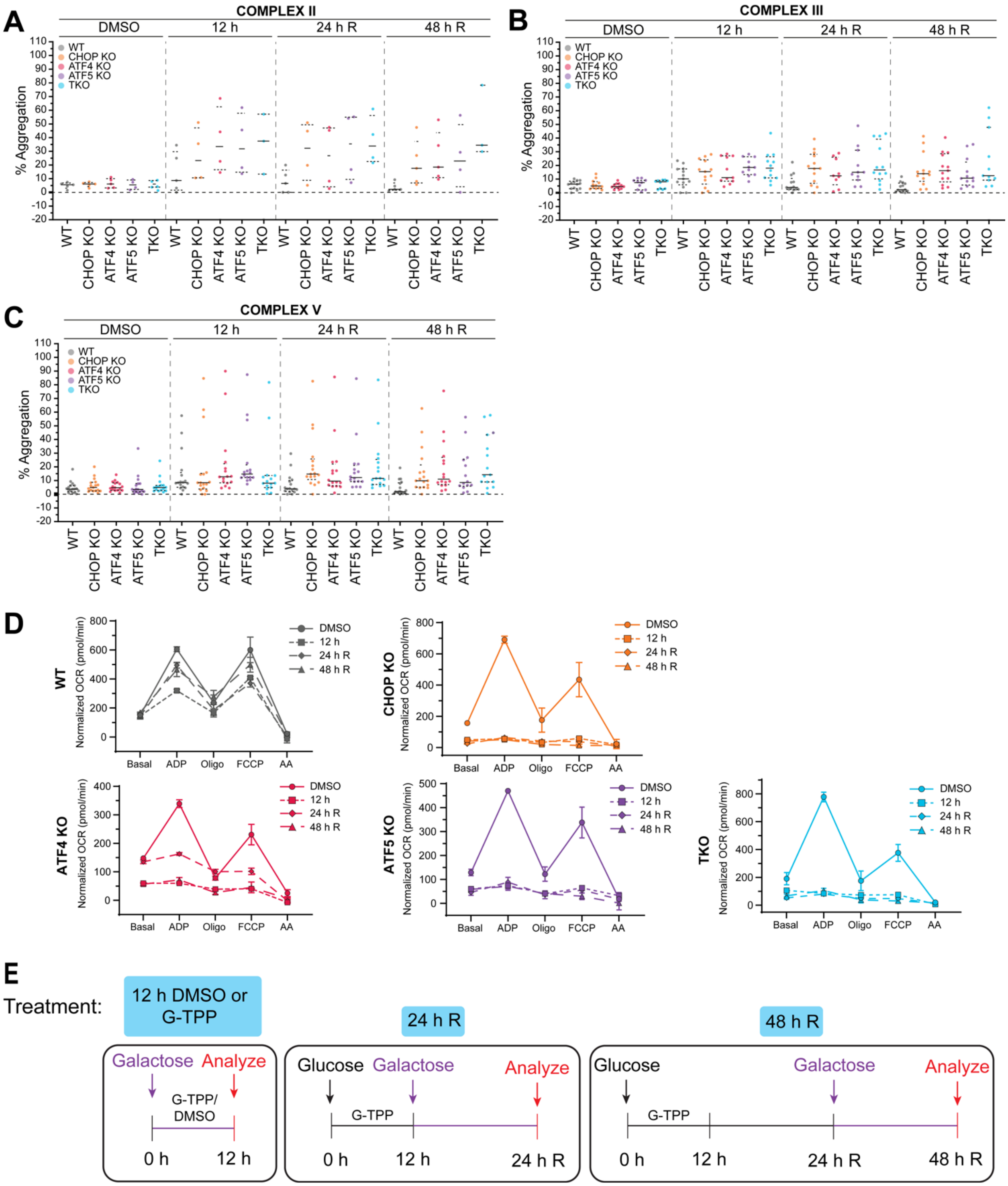

Fig S4. Each CHOP, ATF4 and ATF5-driven signaling arm is required for UPR^{mt}-mediated OXPHOS function and protein solubility protection and repair, related to Fig 4. (**A – C**), Violin plots of the mean % aggregation of proteins comprising complex II (A), complex III (B) and complex V (C) in WT, CHOP KO, ATF4 KO, ATF5 KO and TKO cells at the indicated timepoints. (**D**) Oxygen consumption rates (OCR; pmol/min) of mitochondria isolated from WT, CHOP KO, ATF5 KO, ATF4 KO and TKO samples used to calculate total respiratory capacity

and spare respiratory capacity values in Fig 4. (**E**) Experimental overview of how the Galactose ATP measurements in Figure 4 were performed. Data in (A – D) represent mean data ± SD (D) from three independent experiments.

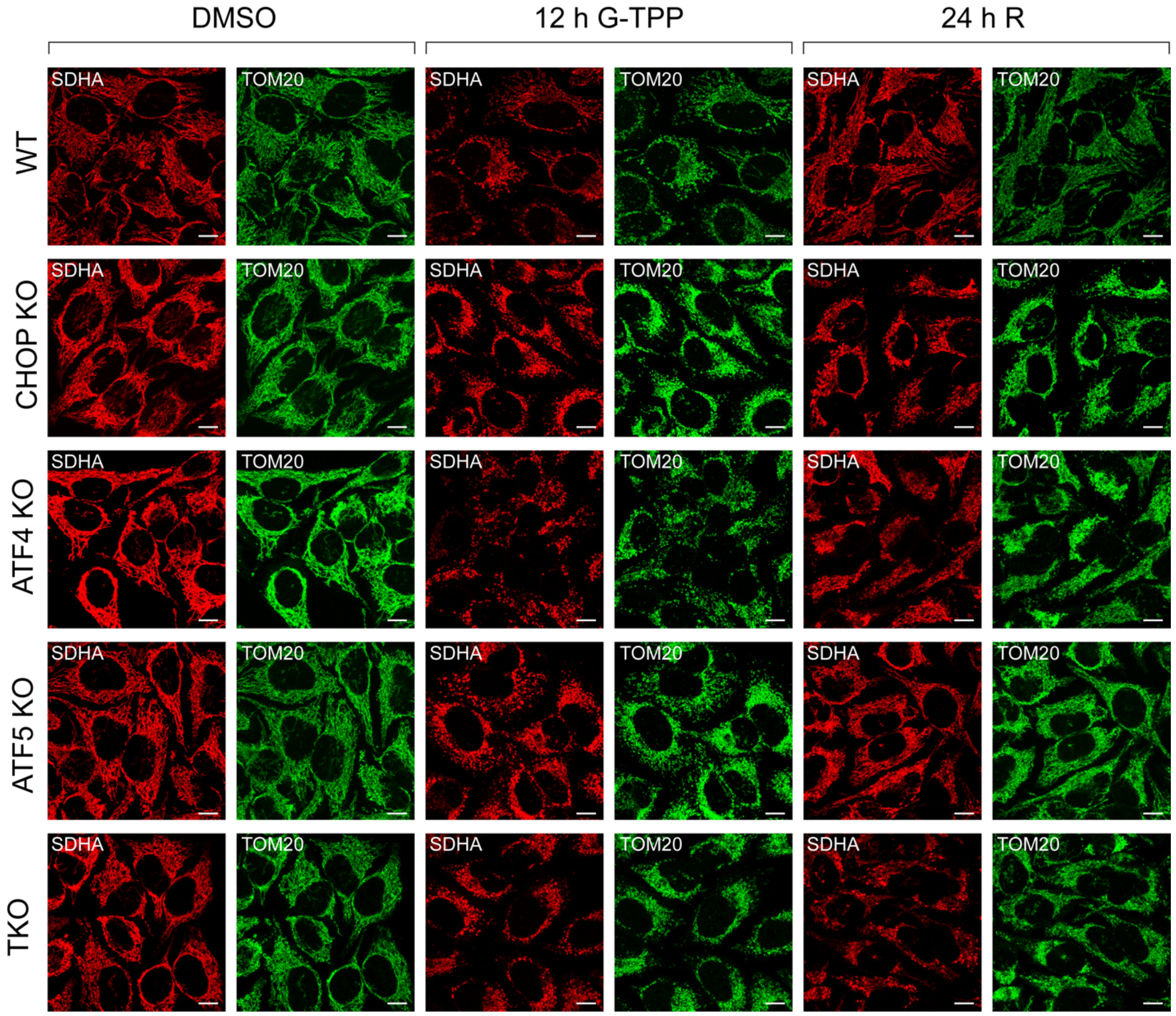

Fig S5. CHOP, ATF4 and ATF5 are required for the restoration of mitochondrial networks following proteostasis stress, related to Figure 5. Representative individual TOMM20 and SDHA immunostaining channels, related to Figure 5. Scale bars = 10 µm.

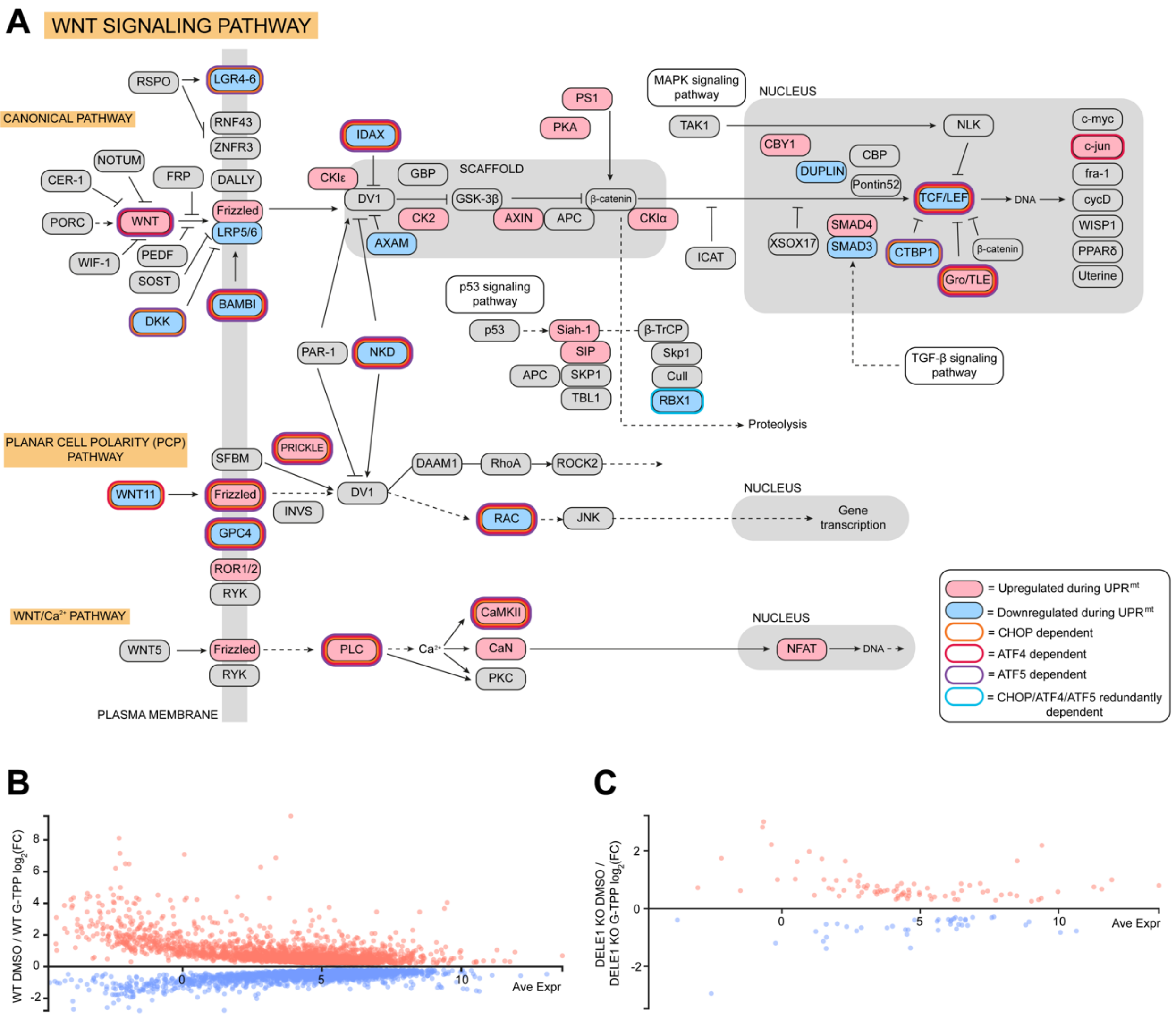

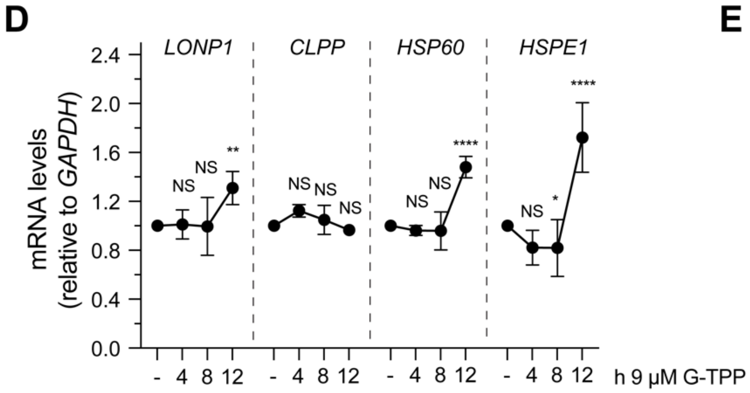

E

GENE	Fold change	GENE	Fold change
HSP90AA1	0.92	*HSPB8*	0.84
HSP90AA2P	0.81	*HSPBAP1*	0.89
HSP90AA6P	1.60	*HSPD1*	0.37
HSP90AB1	0.32	*HSPD1P1*	0.34
HSP90AB3P	0.35	*HSPD1P6*	0.91
HSPA12A	-0.37	*HSPH1*	1.53
HSPA13	0.70	*LONP1*	0.55
HSPA14	0.27	*DNAJA1*	0.58
HSPA1A	3.67	*DNAJA3*	0.45
HSPA1B	3.46	*DNAJA4*	2.09
HSPA1L	1.94	*DNAJB1*	4.05
HSPA2	-0.61	*DNAJB1P1*	4.01
HSPA4	0.45	*DNAJB2*	1.43
HSPA4L	0.88	*DNAJB4*	0.75
HSPA6	9.51	*DNAJB5*	0.49
HSPA7	6.49	*DNAJB6*	0.82
HSPA8	0.36	*DNAJB9*	2.60
HSPA8P4	-1.18	*DNAJC16*	0.50
HSPA9P1	0.44	*DNAJC22*	-0.50
HSPB1	1.01	*DNAJC3-DT*	0.87
HSPB1P1	1.27	*DNAJC7*	0.38
HSPB1P2	1.29	*DNAJC8*	-0.32
HSPB3	-1.20	*ATAD1*	0.45
HSPB7	-0.64		

Fig S6. UPR^{mt} signaling regulated by CHOP, ATF4 and ATF5 drives modulation of Wnt-signaling pathway gene expression during proteostasis stress, related to Fig 6. (**A**) Wnt signaling pathway gene relationships were mapped and UPR^{mt}-related expression changes including transcription factor dependency were annotated on affected genes. (**B**, **C**) Significant gene expression changes in G-TPP treated samples relative to DMSO treated samples in WT (B) and DELE1 KO (C) samples were graphed. (D) mRNA levels relative to GAPDH in WT cells were analyzed for LONP1, CLPP, mitochondrial HSP60 and HSPE1 at the indicated treatment timepoints. (**E**) Selected chaperones and proteases from the transcriptomic data in Figure 6 (both cellular and mitochondrial specific) have been tabulated, including their fold change in WT 12 h G-TPP treated samples relative to DMSO levels. Data in (A – E) represents mean data from three independent experiments.

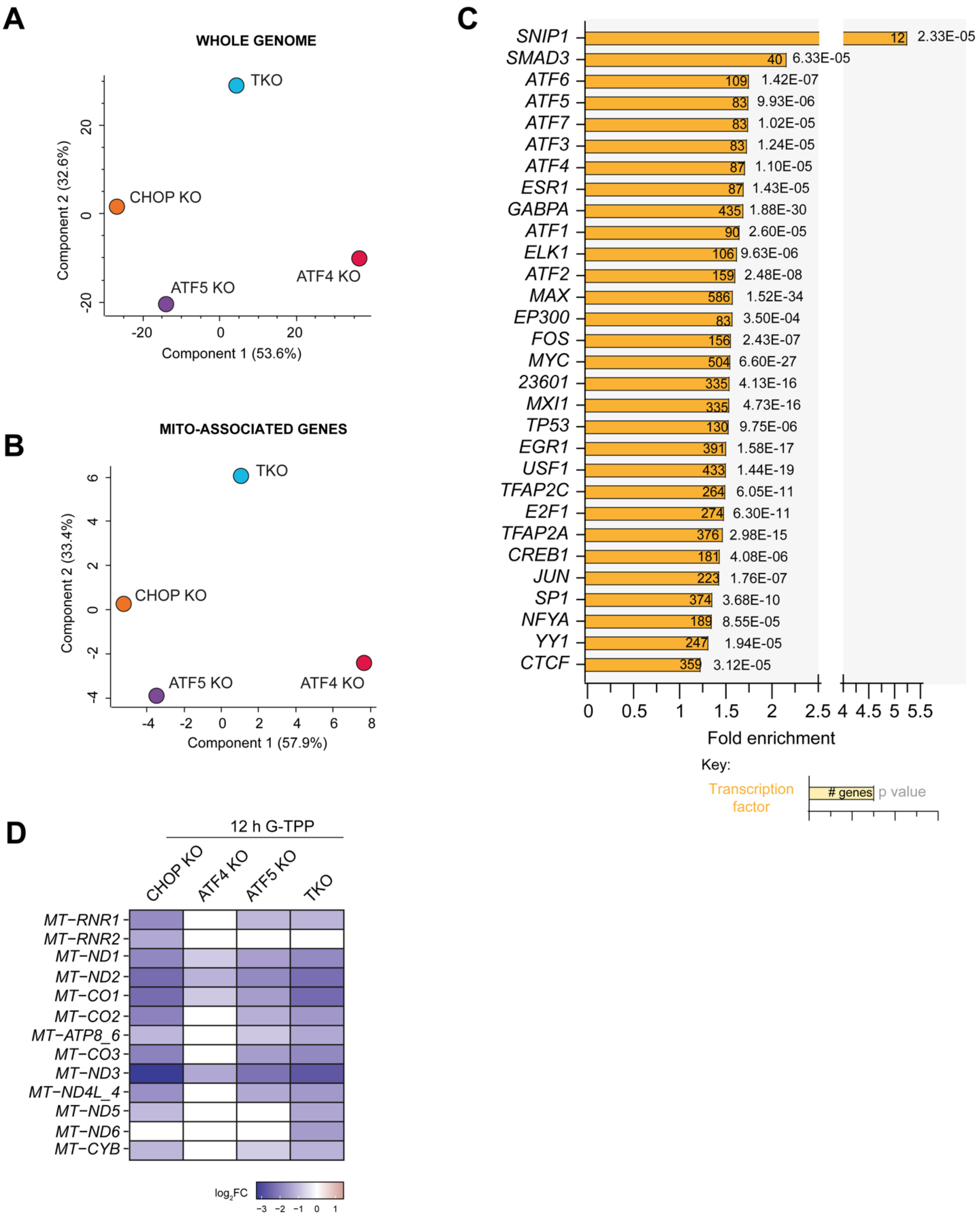

Fig S7. ~44% of the UPR^{mt}-regulated transcriptome is under the regulatory control of unidentified signaling elements, related to Figs 5 and 6. (**A**, **B**) UPR^{mt} gene expression trends across the cellular genome (A) or mitochondrial genome (B) in CHOP KO, ATF4 KO, ATF5 KO

and TKO cells were analyzed by Principal Component Analysis (PCA). (**C**) Genes that did not show reduced expression in any CHOP KO, ATF4 KO, ATF5 KO or TKO transcriptome samples were classified to be under undefined regulatory control. The gene subset with undefined regulatory control was analyzed using the RegNetwork database(*41*) through ShinyGO v0.75(*72*) to identify transcription factors with enriched pathway and signaling representation in the undefined regulatory gene subset. (**D**) Changes in mtDNA-encoded rRNAs and mRNAs levels (expressed as $\log_2$ fold change (FC) of counts per million mapped) were determined by RNA-seq. The expression profiles of each gene showing statistically significant increases are in red and decreases in blue relative to WT controls for the respective treatments; non-significant changing genes are in white. Data in (A), (B) and (D) represents data from three independent experiments. Data in (C) was generated using mean data from three independent experiments.

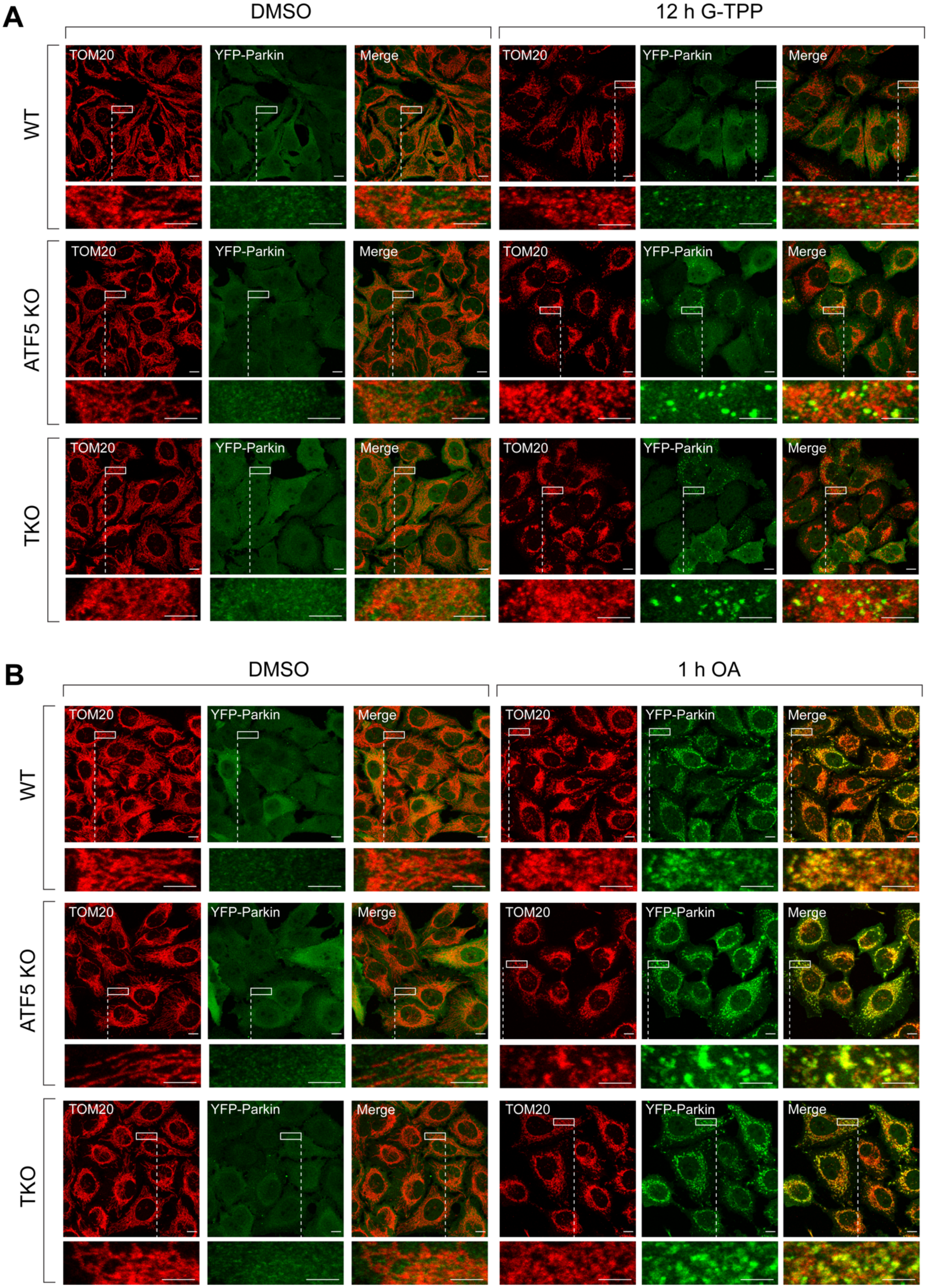

A
DMSO
12 h G-TPP
WT
ATF5 KO
TKO
TOM20
YFP-Parkin
Merge
B
DMSO
1 h OA
WT
ATF5 KO
TKO
TOM20
YFP-Parkin
Merge

Fig S8. **Proteostasis stress induces piecemeal PINK1/Parkin mitophagy, with similar levels of YFP-Parkin recruitment observed in ATF5 KO and TKO cells.** (A-B) WT, ATF5 KO and TKO cells expressing YFP-Parkin were treated for 1 h with DMSO or Oligomycin/Antimycin A (OA) or for 12 h with DMSO or 9 μM G-TPP. Samples were immunostained for TOMM20 and YFP. Overview scale bars = 10 μM, zoom in scale bars = 5 μm.

	Relative to WT				Relative to TKO		
	CHOP KO	ATF4 KO	ATF5 KO	TKO	CHOP KO	ATF4 KO	ATF5 KO
**Amino acid metabolism**	NS	NS	NS	**	NS	NS	NS
**Apoptosis**	NS	NS	NS	NS	NS	NS	NS
**Calcium Signaling and Transport**	NS	NS	NS	NS	NS	NS	NS
**Cardiolipin biosynthesis**	NS	NS	**	NS	NS	NS	*
**Fatty Acid Biosynthesis & Elongation**	NS	NS	*	***	**	**	*
**Fatty Acid Degradation & Beta-oxidation**	NS	NS	NS	**	NS	NS	NS
**Fatty Acid Metabolism**	*	**	*	**	NS	NS	NS
**Fe-S Cluster Biosynthesis**	NS	NS	NS	NS	NS	NS	NS
**Folate & Pterin Metabolism**	NS	NS	NS	NS	NS	NS	NS
**Glycolysis**	NS	NS	NS	*	NS	**	NS
**Heme Biosynthesis**	NS	NS	NS	*	NS	NS	NS
**Import & Sorting**	**	**	*	**	NS	NS	NS
**Metabolism of Lipids & Lipoproteins**	NS	*	*	*	NS	NS	NS
**Metabolism of Vitamins & Co-Factors**	NS	NS	NS	*	NS	NS	NS
**Mitochondrial Carrier**	NS	NS	NS	NS	NS	NS	NS
**Mitochondrial Dynamics**	NS	NS	NS	NS	NS	NS	NS
**Mitochondrial Signaling**	NS	NS	NS	NS	NS	NS	NS
**Mitophagy**	NS	NS	*	**	NS	*	NS
**Nucleotide Metabolism**	NS	NS	NS	**	NS	*	NS
**Oxidative Phosphorylation**	*	**	*	***	*	NS	*
**Pentose Phosphate Pathway**	NS	**	NS	NS	NS	**	NS
**Protein Stability & Degradation**	NS	NS	NS	NS	NS	NS	NS
**Pyruvate Metabolism**	NS	NS	NS	NS	NS	NS	NS
**Replication & Transcription**	**	**	**	****	**	**	**
**ROS Defence**	NS	NS	NS	NS	NS	*	NS
**Translation**	***	***	***	****	*	*	**
**Transmembrane Transport**	*	NS	NS	*	NS	NS	NS
**TCA Cycle**	NS	NS	NS	**	NS	*	NS
**Ubiquinone Biosynthesis**	**	***	**	****	***	**	***
**Mito-proteome**	*	NS	*	**	NS	NS	NS

Table S1. Statistical analysis of rate recovery trends across mitochondrial proteome and process groupings. Samples were analyzed relative to WT rate recovery per 24 h (‘Relative to WT’ column) or TKO rate recovery per 24 h (‘Relative to TKO’ column). ****$p \leq 0.0001$,

***p≤0.001, **p≤0.01, *p≤0.05, NS p>0.05 (one-way ANOVA). Data was generated from three independent experiments.

	DMSO				12 h				24 h recovery				48 h recovery			
	CHOP KO	ATF4 KO	ATF5 KO	TKO	CHOP KO	ATF4 KO	ATF5 KO	TKO	CHOP KO	ATF4 KO	ATF5 KO	TKO	CHOP KO	ATF4 KO	ATF5 KO	TKO
**Amino acid metabolism**	NS	NS	NS	NS	****	****	****	****	****	****	****	****	****	****	****	****
**Apoptosis**	NS	NS	NS	NS	****	***	***	****	****	****	***	***	NS	NS	NS	NS
**Calcium Signaling and Transport**	NS	NS	*	NS	**	****	**	***	****	****	****	****	NS	NS	NS	NS
**Cardiolipin biosynthesis**	NS	**	****	***	****	****	****	****	****	****	****	****	****	****	****	****
**Fatty Acid Biosynthesis & Elongation**	NS	NS	****	**	****	****	****	***	****	****	****	****	****	****	****	****
**Fatty Acid Degradation & Beta-oxidation**	NS	NS	NS	NS	****	****	****	****	****	****	****	****	****	****	****	****
**Fatty Acid Metabolism**	NS	*	****	***	****	****	****	****	****	****	****	****	****	****	****	****
**Fe-S Cluster Biosynthesis**	NS	*	NS	*	****	****	****	****	****	****	****	****	****	****	***	****
**Folate & Pterin Metabolism**	NS	NS	****	*	****	****	****	****	****	****	****	****	****	****	****	****
**Glycolysis**	NS	NS	**	NS	*	****	**	*	***	**	**	**	NS	NS	NS	**
**Heme Biosynthesis**	NS	NS	**	NS	****	****	****	****	****	****	****	****	****	****	****	****
**Import & Sorting**	NS	NS	*	NS	NS	**	**	***	****	****	****	****	***	****	**	****
**Metabolism of Lipids & Lipoproteins**	NS	NS	****	**	***	***	****	***	****	****	****	****	**	***	***	***
**Metabolism of Vitamins & Co-Factors**	NS	NS	*	NS	****	****	****	****	****	****	****	****	****	****	****	****
**Mitochondrial Carrier**	NS	NS	NS	NS	***	****	***	**	****	****	***	****	**	****	***	**
**Mitochondrial Dynamics**	NS	NS	NS	NS	***	****	****	***	****	*	****	***	*	NS	NS	**
**Mitochondrial Signaling**	NS	**	**	***	****	****	****	****	****	****	****	****	***	***	****	****
**Mitophagy**	****	****	NS	NS	NS	*	NS	NS	****	**	****	****	*	NS	**	****
**Nucleotide Metabolism**	NS	NS	NS	NS	****	****	****	****	****	****	****	****	****	****	****	****
**Oxidative Phosphorylation**	NS	NS	NS	NS	***	***	***	**	****	****	****	****	****	****	****	****
**Pentose Phosphate Pathway**	NS	NS	*	**	***	****	***	****	**	****	****	*	NS	*	**	****
**Protein Stability & Degradation**	NS	NS	**	NS	****	****	****	****	****	****	****	****	****	****	****	****
**Pyruvate Metabolism**	**	NS	**	NS	****	****	****	****	****	****	****	****	****	****	****	****
**Replication & Transcription**	NS	NS	**	*	****	****	****	****	****	****	****	****	****	****	****	****
**ROS Defence**	NS	NS	NS	NS	****	****	****	****	****	****	****	****	**	**	*	****
**Translation**	NS	NS	NS	NS	****	****	****	****	****	****	****	****	****	****	****	****
**Transmembrane Transport**	NS	NS	NS	NS	NS	***	**	**	****	NS	***	****	**	NS	*	***
**TCA Cycle**	NS	NS	NS	NS	****	****	****	****	****	****	****	****	****	****	****	****
**Ubiquinone Biosynthesis**	NS	NS	NS	NS	****	****	****	****	****	****	****	****	****	****	****	****
**Mito-proteome**	NS	NS	*	NS	**	***	**	**	***	***	**	***	**	***	***	*

Table S2. Statistical analysis of AI trends across mitochondrial proteome and process groupings. Samples were analyzed relative to WT aggregation index (AI) levels at each time

point. ****$p \leq 0.0001$, ***$p \leq 0.001$, **$p \leq 0.01$, *$p \leq 0.05$, NS $p > 0.05$ (two-way ANOVA). Data was generated from three independent experiments.

Gene Symbol	Uniprot	GeneID /Location	Targeting location	TALEN or CRISPR gRNA (PAM)	Clone number used	Depth/detected Alleles	Mutation	Protein impact
CHOP	P35638	1649/NC_000012.12	Exon 1	Left arm: TCCTCATACCAGGCTTCC Right arm: TCCCGAAGGAGAAAGGCA	#2	10/2	c.[110_111insAACAACAC];c.[110_115delinsGGGTTAGGGTTAGGGTTAGGGTTAGGGTTAGGGTTAGGGTTAGGGTTAGGGTTAGGGTTAGGGTTAGGGTTAGGGTTAGGCCCTAGGGTTAGGGTTTTTTT]	p.[Y61Tfs*29];p.[T60Rfs*31]
CHOP	P35638	1649/NC_000012.12	Exon 1	TTCACCTCCTGGAAATGAAGAGG	#15	NA/4	c.[122_126del]; c.[117_123del]; c.[118_134del]; c.[122_131del]	p.[P42Efs*61]; p.[P41Wfs*43]; p.[S40*]; p.[P42Efs]
ATF4	P18848	468/NC_000022.11	Exon 2	TTTGCAGAGGATGCCTTCTCCGG	#17	NA/4	c.[236_237insN];c.[231_244del];c.[234_247del];c.[237_250del]	p.[F79*26];p.[A78Rfs*22];p.[F79Lfs*21];p.[F79Lfs*21]
ATF5	Q9Y2D1	22809/NC_000019.10	Exon 1	CATGTCACTCCTGGCGACCCTGG	#17	10/3	c.[16_17insG];c.[17_28del];c.[13_51del]	p.[T6Sfs*19];p.[T6K,L7_E10del];p.[A5_P17del]
ATF5	Q9Y2D1	22809/NC_000019.10	Exon 1	CATGTCACTCCTGGCGACCCTGG	#26	NA/1	c.[18del]	p.[T6defs1]
DELE1	Q14154	9812/ NC_000005.10	Exon 2	AGGCTAGGTCCCAGTGTACGGG	#16	NA/4	c.[41_44del];c.[42_43del];c.[43_44del];c.[44_45del]	p.[R14Hfs*22];p.[L16Gfs*18];p.[L16Gfs*18];p.[L16Gfs*18]
				CHOP/ATF4/ATF5 TKO				
ATF5	Q9Y2D1	22809/NC_000019.10	Exon 1	CATGTCACTCCTGGCGACCCTGG	#22 (made from *CHOP* KO #1)	10/2	c.[16del];c.[13_22del]	p.[T6Pfs*25];p.[A5_L7del]
ATF4	P18848	468/NC_000022.11	Exon 2	TTTGCAGAGGATGCCTTCTCCGG	#1 (made from *CHOP/ATF5* DKO #22)	NA/5	c.[227+5_243del];c.[227+5_236del];c.[237_238del];c.[227+4_244del]; c.[227+4_237del]	p.[?](affecting splicing);p.[?](affecting splicing);p.[S80Rfs*24]; p.[?](affecting splicing);p.[?](affecting splicing)

Table S3. CRISPR and TALEN sequences and genotyping results of the knockout cell lines generated in this study. The indels for the targeted genes detected in the indicated knockout cell lines ("Mutation" column) and their translated proteins ("Protein impact" column) are formatted according to Human Genome Variation Society (HGVS; http://varnomen.hgvs.org/). Mutation positions within genes which have multiple splice variants are determined using the variant indicated as the canonical isoform in Uniprot. del = deletion; ins = insertion; c. = coding DNA; p. = protein; fs = frame shift; * = stop codon; [?] = affecting splicing; N= any nucleotide (A, C, T and G); X denotes one of the three amino acids A, T or S. The numbers following the asterisks denote the numbers of amino acids between the first amino acid changed after the mutation(s) and the first subsequent stop codon encountered.

Gene	*Exon*	*Primer*	*Primer sequence (5' to 3')*	*PCR product (bp)*
CHOP	1	Forward	GACCATCCTCTAGACTGCCGGATCCGGATGG CAGCTGAGTCATTG	650
		Reverse	GCGAGATTATAGAGATCCCAAGCTTTCATGC TTGGTGCAGATTC	
ATF4	2	Forward	ATGCGGATCCCTCGATTCCAGCAAAGCACC	742
		Reverse	ATGCAAGCTTGCAGTGTAGTCTGGCTTCC	
		Sequencing	TGAGTGGGCCACCACCACATC	N/A
ATF5	1	Forward	GCGCGGATCCGTAGGTCTTCCACTTTCGCCTT	338
		Reverse	GCGCAAGCTTCTTCCACCTGCCCTTACCT	
DELE1	2	Forward	GGAGTAGGCATAGCCAGTGAAGAG	664
		Reverse	TAGCAGGGTGTGGTGACGGGTG	
		Sequencing	CAGCAGAATCACATGGGC	N/A

Table S4. Genotyping primers for sequencing analysis of the generated knockout cell lines.

Antibodies	Company	Species	Catalog #/RRID
CHOP	Cell Signaling	Mouse	2895/AB_2089254
ATF5	Santa Cruz	Mouse	sc-377168/AB_2943039
ATF4	Santa Cruz	Mouse	sc-390063/AB_2810998
PINK1	Cell Signaling	Rabbit	6946/AB_11179069
p62	Abnova	Mouse	H00008878-M0/AB_4370851
Actin	Cell Signaling	Mouse	3700/AB_2242334
PGAM5	N/A	Rabbit	In house/NA
LRPPRC	Abcam	Rabbit	ab97505/AB_10688419
AARS2	Abcam	Rabbit	ab197367/AB_2943036
SHMT2	Abcam	Rabbit	ab180786/AB_2943037
VDAC1	Cell Signaling	Rabbit	4661/AB_10557420
TOMM20	Santa Cruz	Mouse	sc-17764/AB_628381
TOMM20	Abcam	Rat	ab289670/AB_2943038
Parkin	Santa Cruz	Mouse	sc-32282/AB_628104
VCP	Santa Cruz	Mouse	sc-133212/AB_2214654
ATP5A	Abcam	Mouse	ab14748/AB_301447
NDUFS1	Proteintech	Rabbit	12444-1-AP/AB_2282657
SDHA	Abcam	Mouse	ab14715/AB_301433
anti-GFP	Invitrogen	Chicken	A10262/AB_2534023
AlexaFluor-488 (Anti-Chicken)	ThermoFisher	Goat	A11039/AB_2534096
AlexaFluor-488 (Anti-Rat)	Abcam	Goat	ab150157/AB_2722511
AlexaFluor-555 (Anti-Mouse)	ThermoFisher	Goat	A21422/AB_2535844
AlexaFluor-555 (Anti-Rat)	ThermoFisher	Goat	A21434/AB_2535855
Anti-Mouse IgG, HRP-linked	Cell Signaling	Horse	7076/AB_330924
Anti-Rabbit IgG, HRP-linked	Cell Signalling	Goat	7074/AB_2099233

Table S5. Antibodies used in this study.

Elution step	% ACN	% TEA (0.1%)
Wash	5	95
1	9	91
2	13	87
3	17	83
4	21	79
5	25	75
6	29	71
7	33	67
8	37	63
9	41	59
10	45	55
11	50	50
12	55	45

Table S6. Modified high-pH elution gradient used in TMT batch sample fractionation.

Gene	Primer	Primer sequence (5' to 3')
CHOP	Forward	AGCCAAAATCAGAGCTGGAA
	Reverse	TGGATCAGTCTGGAAAAGCA
ATF4	Forward	CAGCAAGGAGGATGCCTTCT
	Reverse	CCAACAGGGCATCCAAGTC
ATF5	Forward	AAGTCGGCGGCTCTGAGGTA
	Reverse	GGACTCTGCCCGTTCCTTCA
LONP1	Forward	CCCGCGCTTTATCAAGATT
	Reverse	AGAAAGACGCCGACATAAGG
CLPP	Forward	CGTATCATGATCCACCAGCCCTC
	Reverse	CCATGGCGGACTCGATCACCTG
HSP10	Forward	AGTAGTCGCTGTTGGATCGG
	Reverse	TGCCTCCATATTCTGGGAGA
HSP60	Forward	CTGCCACAACCTGAAGACCAAC
	Reverse	ATTGCCAATGCTCACCGTAAGCC
GAPDH	Forward	TGACAACTTTGGTATCGTGGAAGG
	Reverse	AGGCAGGGATGATGTTCTGGAGAG

Table S7. qRT-PCR primers used in this study.

Table S8. TMT-batch layouts, MitoPQ data of WT 12 h G-TPP treatment samples (related to Fig 1) and WT, CHOP KO, ATF4 KO, ATF5 KO and TKO proteostasis stress and recovery samples (Related to Figs 2, 3, 4). (Provided as a separate .xlsx file)

Table S9. NGS transcriptome data of WT, CHOP KO, ATF4 KO, ATF5 KO and TKO 12 h DMSO or 12 h G-TPP treated samples (related to Figs 6, 7). (Provided as a separate .xlsx file)
